# MicroRNA-18a inhibits hypoxia-inducible factor 1α activity and lung metastasis in basal breast cancers

**DOI:** 10.1186/bcr3693

**Published:** 2014-07-28

**Authors:** Raisa Krutilina, Wenlin Sun, Aarti Sethuraman, Martin Brown, Tiffany N Seagroves, Lawrence M Pfeffer, Tatyana Ignatova, Meiyun Fan

**Affiliations:** 1 Departments of Pathology and Laboratory Medicine, University of Tennessee Health Science Center, 19 South Manassas Street, Memphis, TN 38163 USA; 2 Center for Cancer Research, University of Tennessee Health Science Center, 19 South Manassas Street, Memphis, TN 38163 USA; 3 Department of Pharmacology, University of Tennessee Health Science Center, 424 Crowe research building, 874 Union Avenue, Memphis, TN 38163 USA; 4 Department of Neurosurgery, University of Tennessee Health Science Center, 847 Monroe Avenue, Memphis, TN 38163 USA

## Abstract

**Introduction:**

In breast cancer, distinct expression profiles of microRNAs (miRNAs) have been associated with molecular subgroups and clinicopathological characteristics, implicating a diagnostic and prognostic role of miRNAs. However, the biological functions of deregulated miRNAs in tumor progression are not yet completely defined. In this study, we investigated the function of miR-18a in regulating breast cancer metastasis through the hypoxia-inducible factor 1α (HIF1A)–dependent hypoxic response.

**Methods:**

An orthotopic metastatic breast cancer xenograft model (MDA-MB-231 cells) was used to identify miRNAs associated with spontaneous lung metastasis. The function of miR-18a in regulating HIF1A expression, as well as cellular responses to hypoxia and metastasis, were then studied *in vitro* and *in vivo* by assessing ectopic miR-18a expression or miR-18a inhibition. miRNA–mRNA interactions (AGO2 immunoprecipitation and 3′ untranslated region Luciferase reporter assays), gene expression (quantitative PCR and microarray), cell migration and invasion, and cell growth were assessed under normoxic or hypoxic conditions, complemented by orthotopic xenograft of tumor cells to the mammary fat pad to investigate the effect of modulating miR-18a expression on primary tumor growth and lung metastasis. Last, clinically relevant correlations between miR-18a, HIF1A, hypoxia-responsive gene expression and distant metastasis–free survival (DMFS) were assessed using published expression array breast tumors data sets.

**Results:**

miRNAs encoded by the *MIR17HG* gene were downregulated in lung metastases compared to primary tumors. Ectopic expression of miR-18a, a *MIR17HG* family member, in a metastatic variant of MDA-MB-231 cells reduced primary tumor growth and lung metastasis, whereas miR-18a inhibition in the parental cells promoted tumor growth and lung metastasis. We identified HIF1A as a direct target of miR-18a. Modulating miR-18a expression significantly affected hypoxic gene expression, cell invasiveness and sensitivity to anoikis and hypoxia *in vitro* in a HIF1A-dependent manner. Analysis of previously published data revealed that higher expression of HIF1A and a panel of hypoxic genes is associated with shorter DMFS interval in patients with basal-like breast tumors, and that, within this subtype, miR-18a expression is inversely correlated with hypoxic gene expression. Together, these data support a role of miR-18a in repressing distant metastasis through a HIF1A-dependent pathway.

**Conclusions:**

The results of this study reveal a novel role for miR-18a in targeting HIF1A and repressing metastasis of basal-like breast tumors.

**Electronic supplementary material:**

The online version of this article (doi:10.1186/bcr3693) contains supplementary material, which is available to authorized users.

## Introduction

MicroRNAs (miRNAs) play an important role in coordinating spatial and temporal expression of proteins by regulating mRNA translation and stability [1]. Deregulation of miRNAs has been linked to tumor development and progression, and a growing number of miRNAs have been described as candidate oncogenes or tumor suppressors [2]. In breast cancer, distinct expression profiles of miRNAs have been associated with specific molecular subtypes and clinicopathological characteristics, implicating a diagnostic and prognostic role of miRNAs [3–5]. However, the biological functions of the deregulated miRNAs in tumor progression have not yet been completely defined.

One of the most frequently deregulated miRNA-encoding genes in human cancer is the polycistronic *MIR17HG* gene, which encodes six miRNAs (miR-17, miR-20a, miR-18a, miR-19a, miR-19b and miR-92a) [6]. *MIR17HG* was originally described as an oncomir because of its oncogenic function in the hematological system, thyroid and lung [7]. However, emerging evidence suggests that loss of function of *MIR17HG* might contribute to the development and progression of other types of cancers, implicating a tumor suppressor function. For example, loss of heterozygosity at chromosome 13q31, where the human *MIR17HG* gene is located, was detected in approximately 25% of human breast tumors [8]. In addition, *MIR17HG* overexpression was found to inhibit proliferation of luminal breast cancer cells by targeting a steroid receptor coactivator (*NCOA3*), cyclin D1 (*CCND1*) and estrogen receptor α (*ESR1*) [9–12]. Furthermore, distinct physiological functions have been linked to the different miRNA members encoded by the *MIR17HG* gene [10, 13, 14]. Therefore, to gain a more complete understanding of the physiological impact of *MIR17HG* deregulation in cancer, a detailed investigation of each individual *MIR17HG* family member in multiple types of tumor cells is required.

In this study, we discovered that, compared to parental cells (MB231RN) or a subline derived from the primary tumors (MB231RN-MFP), miRNAs encoded by *MIR17HG* were downregulated in a MDA-MB-231 subline isolated from spontaneous lung metastases (MB231RN-LM) and generated from tumor cells orthotopically implanted in the mammary fat pad. Functional studies of miR-18a, a relatively understudied *MIR17HG* family member, revealed a major role in limiting continuous tumor growth and suppressing tumor metastasis, in part by direct regulation of hypoxia-inducible factor 1α (HIF1A) activity. Analysis of previously published expression data revealed that higher expression of HIF1A and a panel of hypoxic genes is associated with a shorter interval of distant metastasis–free survival (DMFS) only in basal-like breast tumors. Additionally, a significant inverse correlation between miR-18a expression and hypoxic gene expression was discovered in basal-like tumors. These data suggest that downregulation of miR-18a and concomitant upregulation of HIF1A activity may be essential to promoting basal breast cancer metastasis to distant organs, including the lungs.

## Material and methods

### Cell culture and stable transfection

MDA-MB-231, MCF7 and MDA-MB-436 cells (American Type Culture Collection, Manassas, VA, USA) were maintained in minimal essential medium supplemented with 100 U/ml penicillin, 100 μg/ml streptomycin and 10% fetal bovine serum. To facilitate tumor imaging and tumor cell isolation *in vitro*, MDA-MB-231 cells were stably transfected with pEF-mRFP [15] and pReceiver-Lv151 (GeneCopoeia, Rockville, MD, USA). The transfected cells that express red fluorescent protein (RFP) and neomycin-resistant marker were designated MB231RN and were used to derive other sublines used in this study. To establish cells that constitutively express miR-18a, lung metastatic variants of MDA-MB-231 (LM) cells were transduced with pEZX-MR06 (GeneCopoeia), a lentiviral vector that encodes a miR-18a stem loop and green fluorescent protein (GFP), and were sorted to obtain GFP-positive cells (LM-miR-18a). Control cells were transduced with pEZX-MR03 (GeneCopoeia) expressing GFP only (LM-GFP). The miArrest miR-18a inhibitor encoded by a lentiviral vector that coexpresses mCherry (HmiR-AN0255-AM03; GeneCopoeia) was used to establish MDA-MB-231 sublines with low miR-18a activity (MB231-18aIN). Control cells were transduced with pEZX-AM03 (GeneCopoeia) expressing mCherry only (MB231-C). HIF1A knockdown was achieved by transducing MB231-C or MB231-18aIN cells with lentiviruses expressing a short-hairpin RNA (shRNA) to *HIF1A* (pLKO.1-HIF1AshRNA: NM_001530.x-1048s1c1; Open Biosystems/GE Dharmacon, Lafayette, CO, USA).

### Generating cell sublines from spontaneous lung metastases and orthotopic primary tumors

MB231RN cells (5 × 10^5^ cells/10 μl phosphate-buffered saline) were surgically inoculated into the right inguinal mammary glands of 4-week-old female NOD *scid* γ mice (NOD.Cg *Prkdc*^*scid*^*Il2rg*^*tm1Wjl*^/SzJ, hereinafter NSG; The Jackson Laboratory, Bar Harbor, ME, USA). Unless otherwise stated, primary tumors were resected in survival surgery performed 4 weeks postinoculation. The lungs were extracted 5 weeks after primary tumor removal and examined for the presence of metastatic loci that express RFP under a fluorescence microscope (CFI60; Nikon Instruments, Melville, NY, USA). To establish the lung metastatic subline, lungs displaying metastases were minced with blades, digested with 1 mg/ml collagenase type III (Worthington Biochemical, Lakewood, NJ, USA) overnight at 37°C, pelleted (1,500 rpm, 5 minutes), washed four times with Dulbecco’s modified Eagle’s medium (DMEM) and plated in gelatin-coated flasks containing DMEM/F-12 medium (Life Technologies, Carlsbad, CA, USA) supplemented with 10% fetal bovine serum, 30% human foreskin fibroblast conditioned medium, 100 U/ml penicillin, 100 μg/ml streptomycin, 10 mg/ml fungin (InvivoGen, San Diego, CA, USA) and 400 μg/ml neomycin. Cells obtained from three lungs were pooled and designated the MB231RN-LM subline. Primary tumors grown in mammary gland fat pads were digested and subjected to culture to obtain the MB231RN-MFP subline. The same experimental procedures were used to generate MB231RN-CE-LM and MB231RN-CE-MFP sublines derived from mice inoculated with a clonal expansion (CE) of a single colony from MB231RN cells (MB231RN-CE). All animal studies adhered to protocols approved by the Institutional Animal Care and Use Committee of the University of Tennessee Health Science Center.

### Primary tumor growth and lung metastasis

To monitor primary tumor growth, mice were inspected weekly by manual palpation for tumor appearance. Tumor growth was monitored twice weekly using digital calipers. Tumor volume was calculated as follows: Volume = (Width^2^ × Length)/2. To examine lung metastases, primary tumors were either surgically removed 4 weeks after inoculation or left intact as indicated, and the lungs were extracted as described per experiment. Metastatic foci of RFP-expressing cells located on the dorsal surface of the left lung lobe were imaged and counted using a fluorescence microscope at × 10 magnification (CFI60; Nikon Instruments). The presence of tumor cells in the left lobes was further confirmed by hematoxylin and eosin (H&E) staining of formalin-fixed lung sections (10 μm thick). In addition, the right lobes of the lungs were digested to prepare DNA, which was then subjected to quantitative PCR (qPCR) analysis using primers specific for the human Alu sequences (hAlu-qPCR), as described previously [16].

### Quantitation of mRNA and miRNA expression by qPCR

Total RNA was prepared using TRIzol reagent (Life Technologies). mRNAs and miRNAs were converted to cDNA using iScript cDNA synthesis kits (Bio-Rad Laboratories, Hercules, CA, USA) and the NCode™ miRNA First-Strand cDNA Synthesis Kit (Life Technologies), respectively. qPCR was performed on the CFX96™ Touch Real-Time PCR Detection System using SYBR Green Supermix (Bio-Rad Laboratories). mRNA and miRNA expression data were normalized to β-actin (*ACTB*) or a small nuclear ribonucleic acid (U6), respectively, using the 2^−ΔΔCt^ method. Primer sequences for mRNAs were obtained from PrimerBank [17], and for miRNAs they were designed according to the manufacturer’s instructions included with the NCode™ miRNA First-Strand cDNA Synthesis Kit. The expression levels of unprocessed transcript of *MIR17HG* (*pri-MIR17HG*) were examined using the TaqMan Pri-miRNA assay (Life Technologies) as described previously [16].

### HIF1α 3′-UTR luciferase reporter assay

Luciferase reporter containing the 3′ untranslated region (3′-UTR) of *HIF1A* mRNA (HIF1A-3′-UTR-Luc, S809216; SwitchGear Genomics, Carlsbad, CA, USA) and pSV-β-galactosidase (Promega, Madison, WI, USA), along with pEZX-MR06 (expressing miR-18a and GFP) or control vector pEZX-MR03 (expressing GFP only), were transfected to MDA-MB-231/DROSHA-shRNA cells [16] using Lipofectamine 2000 reagent (Life Technologies) according to the manufacturer’s instructions. The luciferase and β-galactosidase activities were determined at 48 hours posttransfection using the Luciferase and Β-Glo Assay Systems (Promega), respectively. Reported luciferase activities were normalized to β-galactosidase activities. The Phusion Site-Directed Mutagenesis Kit (Thermo Scientific, Asheville, NC, USA) and the following 5′-phosphorylated primers were used to generate HIF1A-3′-UTR-Luc reporters with mutated or deleted miR-18a binding sites: mutagenic primer, forward 5′-CATTTTAAAAAATGCggCgTTTTATTTATTTATT-3′ and reverse 5′-ATG CTACTGCAATGCAATGGTTTAAATAC-3′; deletion primer forward 5′-TTTATTTATTTTTGGCTAGGGAGTTTATCC-3′ and reverse 5′-TTTTTAAAATGATGCTACTGCAATGC-3′.

### AGO2 immunoprecipitation, immunoblotting and enzyme-linked immunosorbent assay

Argonaute 2 (AGO2) immunoprecipitation was performed as described previously [16]. For immunoblotting, whole-cell lysates were prepared with radioimmunoprecipitation assay buffer (Thermo Scientific), and nuclear extract was prepared as described previously [18]. Cell lysates were resolved in SDS-PAGE, transferred to polyvinylidene fluoride membrane and immunoblotted with the following antibodies: anti-HIF1A (Epitomics, Burlingame, CA, USA), anti-TATA-binding protein (anti-TBP; Santa Cruz Biotechnology, Santa Cruz, CA, USA), anti-PFKFB3 (6-phosphofructo-2-kinase/fructose-2,6-biphosphatase-3; Cell Signaling Technology, Danvers, MA, USA), anti-BHLHE40 (basic helix-loop-helix family, member e40; Abnova, Taipei, Taiwan) and anti-GAPDH (glyceraldehyde-3-phosphate dehydrogenase; EMD Millipore, Billerica, MA, USA). A cell-based enzyme-linked immunosorbent assay (ELISA) (KCB1935; R&D Systems, Minneapolis, MN, USA) that measures total HIF1A protein in the context of whole cells was used to quantify HIF1A protein levels.

### Migration and invasion assays

Cells (2 × 10^4^ cells/0.5 ml/well) were plated onto control membrane inserts with 8-μm pores or onto Matrigel-coated membrane inserts (BD Biosciences, San Diego, CA, USA) that were placed in 24-well chambers filled with 0.6 ml of growth medium. Twenty-four hours after plating, cells that remained on the upper surface of the membrane were removed using cotton-tipped swabs, and cells that had migrated to or invaded the lower surface of the membrane were fixed with methanol, stained with 0.5% crystal violet and counted under the microscope. The migration percentage was calculated as follows: Migration (%) = (Mean number of cells invading through uncoated insert × 100)/Mean number of cells seeded onto the regulator culture surface. The invasion percentage was calculated as follows: Invasion (%) = (Mean number of cells invading through Matrigel insert membrane × 100)/Mean number of cells migrating through control insert membrane.

### Cell growth and viability assays

To examine cell growth, cells were plated at a density of 3 × 10^4^ cells/2 ml/well (six-well dish) and counted daily. To culture cells under lactic acidosis and hypoxic conditions, cells were seeded into medium with 25 mM lactic acid (pH 6.7) and incubated in a Galaxy 14S multigas incubator supplemented with 2% O_2_ and 5% CO_2_. To induce anoikis, cells (5 × 10^4^/well) were seeded into six-well dishes coated with polyhydroxyethylmethacrylate (Sigma-Aldrich, St Louis, MO, USA) to prevent cell attachment. Viable cells were counted using a trypan blue exclusion assay.

### Microarray analysis

To induce chemical hypoxia, cells were treated with 200 μM cobalt(II) chloride (CoCl_2_) for 4 hours. Total RNA from two sets of independent experiments was prepared using the RNeasy Mini Kit (QIAGEN, Valencia, CA, USA) and submitted to the Center of Genomics and Bioinformatics at the University of Tennessee Health Science Center (Memphis, TN, USA) for labeling and hybridization to HT-12 expression BeadChips (Illumina, Chicago, IL, USA). Hybridization signals were processed using Illumina Genome Studio software for annotation, background subtraction, quantile normalization and presence call filtering. The normalized hybridization signals with intensity <40 were set to the flooring value of 40. GeneSpring GX software (Agilent Technologies, Santa Clara, CA, USA) was used for statistical computing. The cutoff parameters for differentially expressed genes were false discovery rate (FDR) = 0.1 and fold change (FC) ≥ 1.5. The differentially expressed genes were further subjected to functional annotation and signaling pathway mapping using QIAGEN’S Ingenuity Pathway Analysis (IPA) (Redwood City, CA, USA). The expression array data have been deposited in the Gene Expression Omnibus (GEO) database [GEO:GSE45362].

### Statistical analysis of correlation between miR-18a and HIF1α expression in breast tumors

The expression data of breast tumor samples were retrieved from the GEO database [GEO:GSE19783, GEO:GSE22220, GEO:GSE28884] or the Cancer Genome Atlas (TCGA). Tumor samples were assigned to five subtypes (luminal A, luminal B, Her2-enriched, basal-like and normal-like) using the PAM50 classifier [19]. The correlation between miR-18a and HIF1A expression was analyzed using GraphPad Prism 5 software (GraphPad Software, La Jolla, CA, USA). The association of HIF1A and hypoxia-responsive genes with distant metastasis was analyzed with the GOBO program (Gene expression-based Outcome for Breast cancer Online) [20].

## Results

### Generation of MDA-MB-231 subline with high metastatic potential

**Figure 1:**
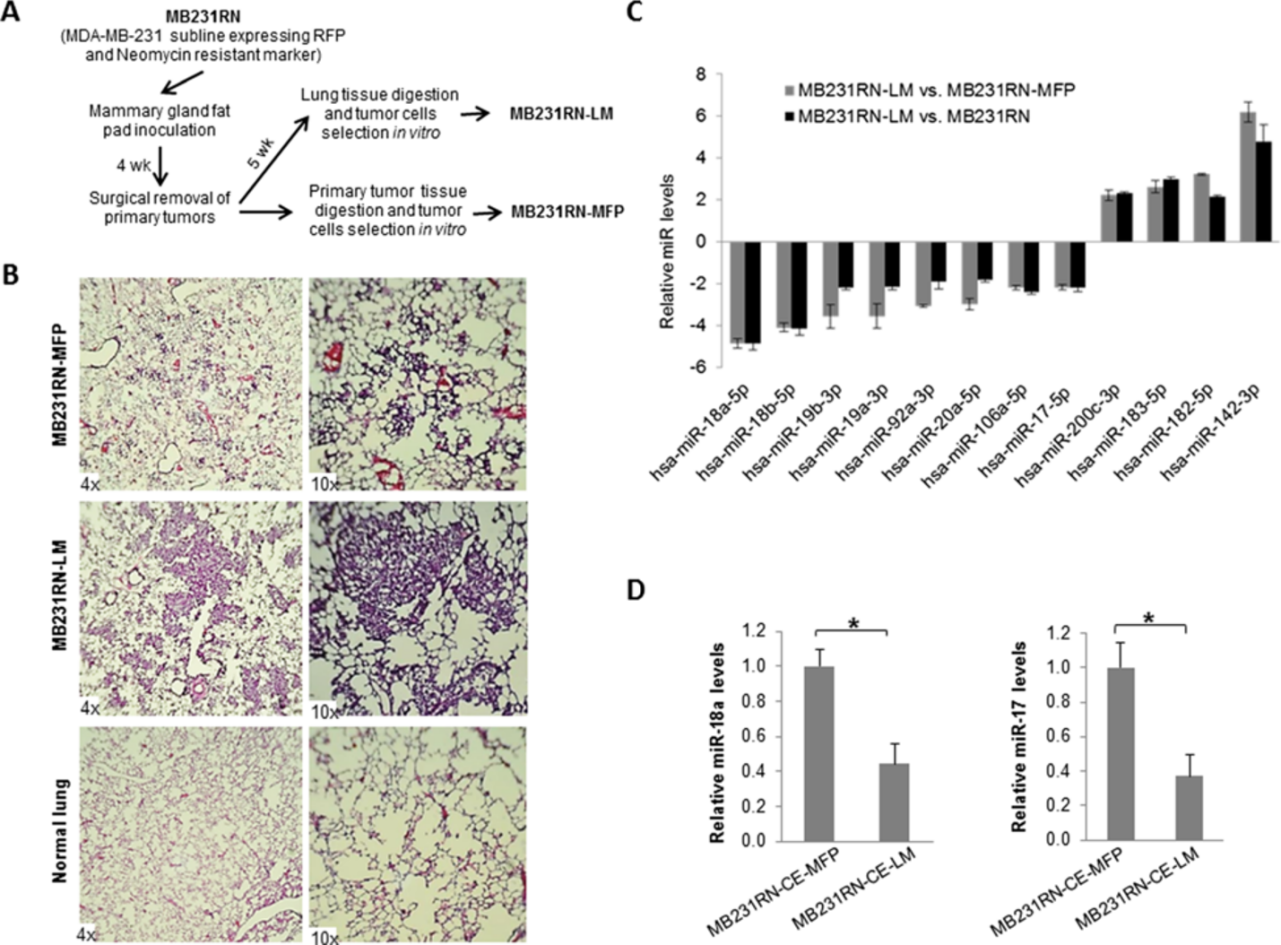
**Identification of microRNAs associated with lung metastasis. (A)** Flowchart outlining procedures used to establish sublines from MB231RN, a MDA-MB-231 variant expressing red fluorescent protein (RFP) and a neomycin resistance marker. The MB231RN-LM subline was isolated from spontaneous lung metastases, whereas the MB231RN-MFP subline was isolated from primary tumors grown in mammary fat pads (MFPs). **(B)** Representative hematoxylin and eosin–stained lung sections from mice killed 7 weeks after tumor cell inoculation (2.5 × 10^5^ cells/injection/mouse, 5 mice/group) are shown. **(C)** Fold changes of microRNAs (miRNAs) differentially expressed in MB231RN-LM cells compared to MB231RN-MFP cells (gray bars) and parental MB231RN cells (black bars). The results are presented as mean ± SD (*n* = 3). **(D)** Expression of miR-18a and miR-17 in sublines MB231RN-CE-MFP and MB231RN-CE-LM, which were generated from primary tumors (CE-MFP) or lung metastases (CE-LM) harvested from mice inoculated with cells expanded from a single colony of the MB231RN line. Relative expression levels of miRNAs are presented as mean ± SD (*n* = 3). *P < 0.05 by Student’s *t*-test. *n*, the number of experimental repeats.

To identify miRNAs involved in breast cancer metastasis, we generated a lung metastatic subline by using an orthotropic metastatic mouse model of basal-like breast cancer MDA-MB-231 cells. MDA-MB-231 cells that express RFP and the neomycin-resistant marker (MB231RN) were surgically inoculated into the right inguinal mammary gland fat pad of 4-week-old female NSG mice, followed by primary tumor resection 4 weeks later, and lung tissue was extracted at 9 weeks after cell inoculation as shown in Figure 1A. The primary tumor and lung tissues were digested and subjected to *in vitro* culture to obtain the sublines MB231RN-MFP and MB231RN-LM, representing tumor cells grown at the orthotopic site of the mammary fat pad (MFP) or that spontaneously metastasized to lung (LM), respectively. To compare the metastatic potential of the sublines, cells (2.5 × 10^5^/injection) were inoculated into the right inguinal mammary gland fat pad of 4-week-old female NSG mice, and the presence of spontaneous metastatic foci in the lungs was examined by histological analysis at 7 weeks posttransplant. Small lung metastases (micrometastases) were observed in mice inoculated with MB231RN-MFP cells (Figure 1B, top panel). In contrast, large areas of lung parenchyma were occupied by tumor cells (macrometaststases) in all mice (*n* = 5) that received MB231RN-LM cells (Figure 1B, middle panel). These results validate the enhanced lung metastatic potential of the MB231RN-LM subline relative to MB231RN parental cells or MB231RN-MFP cells.

### Downregulation of microRNAs in MB231RN-LM cells

To identify miRNAs involved in metastasis, TaqMan-based miRNA array analysis was performed using MB231RN, MB231RN-LM and MB231RN-MFP cells. Among the 377 human miRNAs examined, 10 were found to be significantly deregulated in lung metastatic MB231RN-LM cells in comparison to MB231RN or MB231RN-MFP cells (six downregulated and four upregulated). The differential expression of these miRNAs was independently confirmed by qPCR (Figure 1C). Notably, all of the downregulated miRNAs in the MB231RN-LM cells are encoded by *MIR17HG*. Examination of unprocessed transcripts showed that the expressed level of *pri-MIR17HG* was significantly reduced in MB231RN-LM cells compared to MB231RN-MFP cells (the number of experimental repeats = 3, FC = 1.54, *P* < 0.05). This result suggests that deregulation of miRNAs encoded by *MIR17HG* in metastatic cells is due to reduced gene transcription rather than to impaired miRNA processing.

To rule out the possibility that the downregulation of *MIR17HG* in lung metastatic cells resulted from selection of a clonal variant present in the pooled MB231RN population that may intrinsically express low levels of *MIR17HG*, we repeated the same *in vivo* experiment using a subline derived from a single MB231RN colony (MB231RN-CE). Tumor cells isolated from spontaneous lung metastases (MB231RN-CE-LM) and orthotopic primary tumors (MB231RN-CE-MFP) using this clonally expanded line were subjected to miRNA expression analysis. The expression of miR-18a, along with miR-17, was significantly decreased in cells derived from lung metastases relative to those derived from primary tumors (Figure 1D). This result confirmed that *MIR17HG* downregulation is an acquired trait associated with spontaneous lung metastasis rather than clonal selection.

### miR-18a suppresses tumor growth and lung metastasis *in vivo*

**Figure 2:**
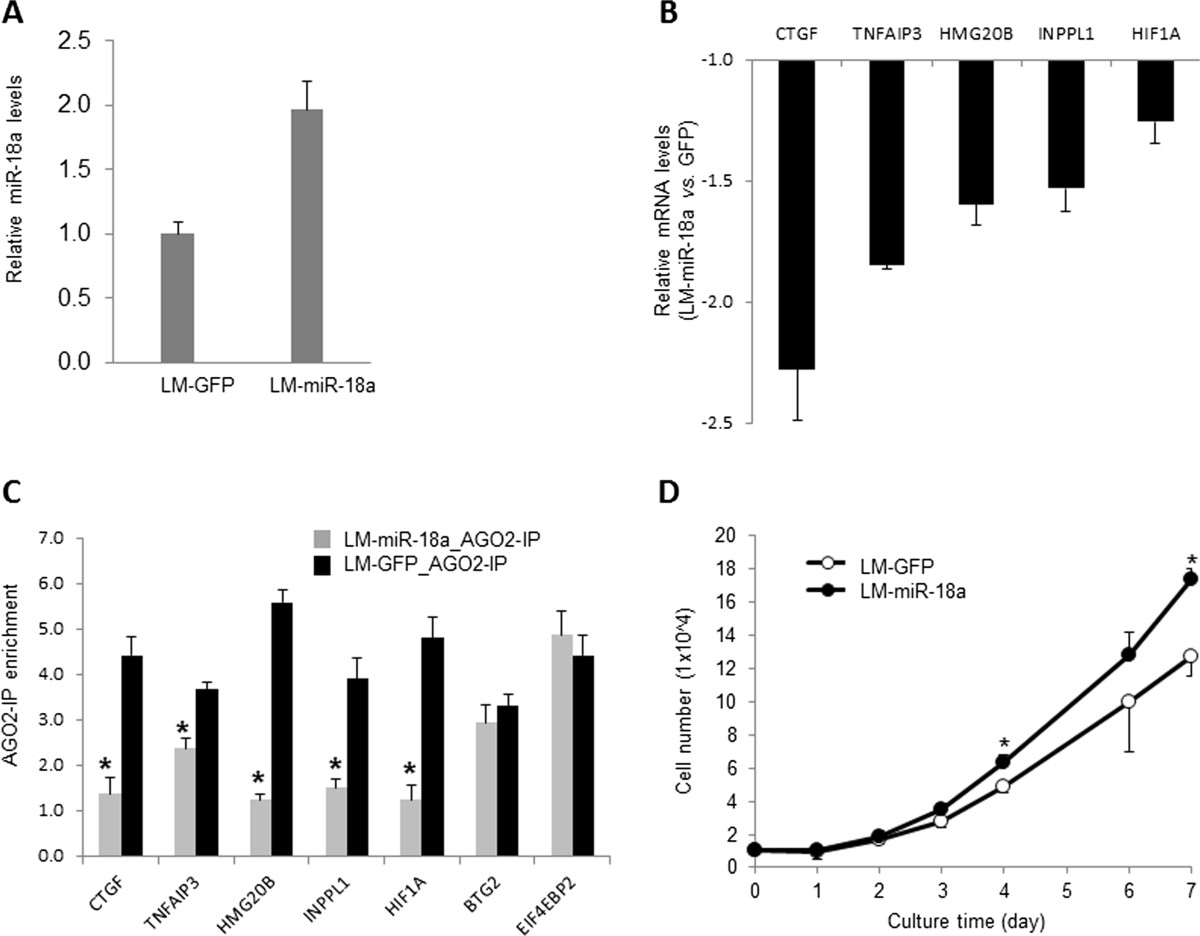
**Effects of ectopic microRNA-18a expression on gene expression and cell growth in MB231RN-LM cells. (A)** Expression levels of microRNA-18a (miR-18a) in cells transduced with lentiviral vector expressing miR-18a green fluorescent protein (GFP) (LM-miR-18a) and or control cells transduced with lentiviral vector expressing GFP only (LM-GFP). **(B)** Effect of miR-18a on expression levels of predicted target mRNAs. mRNA levels were examined by quantitative PCR (qPCR) and normalized to *ACTB*. The expression levels of all the presented genes were significantly reduced by miR-18a expression (*n* = 3, *P* < 0.05 by Student’s *t*-test). **(C)** Effect of miR-18a on RNA-induced silencing complex (RISC) binding of predicted target mRNAs. RISC binding was examined by AGO2 immunoprecipitation (AGO2-IP) followed by RNA purification and qPCR, presented as fold change enrichment (AGO2-IP vs control immunoglobulin G immunoprecipitation). *P < 0.05 by Student’s *t*-test (fold enrichment of AGO2-IP in LM-miR-18a cells vs in control cells). **(D)** Growth curves of LM-miR-18a and LM-GFP cells. All results are presented as mean ± SD (*n* = 3). *P < 0.05 by Kruskal-Wallis analysis of variance followed by Dunn’s multiple-comparisons test).

Compared to other *MIR17HG* family members, miR-18a is understudied and its function in tumor cells is largely undefined. The maturation of miR-18a, but not other *MIR17HG* family members, has been reported to be specifically regulated by heterogeneous nuclear riboprotein A1 (HNRNPA1), implicating tight control of miR-18a abundance in human cells [21]. Therefore, we speculated that miR-18a deregulation might directly impact cell metastatic potential. To examine whether miR-18a regulates primary tumor growth and metastasis, we used a lentiviral construct that encodes a miR-18a stem loop and GFP to ectopically express miR-18a in MB231RN-LM cells (LM-miR-18a). An approximately twofold increase of miR-18a expression was detected by qPCR in the transduced cells, in comparison to cells that were transduced with a control vector expressing GFP only (LM-GFP) (Figure 2A). Because translation inhibition mediated by miRNAs is usually coupled with target mRNA degradation [22], we performed qPCR to examine the mRNA levels of a panel of predicted miR-18a targets to confirm that the ectopically expressed miR-18a functions properly in the transfected cells. Five predicted targets of miR-18a were identified according to TargetScan [23], including connective tissue growth factor (*CTGF*), tumor necrosis factor α–induced protein 3 (*TNFAIP3*), high-mobility group 20B (*HMG20B*), inositol polyphosphate phosphatase-like 1 (*INPPL1*) and *HIF1A*. As shown in Figure 2B, the expression levels of these five mRNAs that harbor conserved binding sites with an exact match to positions 2 through 8 of miRNA-18a in 3′-UTR were significantly decreased (the number of experimental repeats = 3; *P* < 0.05) in response to ectopic miR-18a expression. We next examined the interaction of miR-18a targets with RNA-induced silencing complex (RISC) by using AGO2 immunoprecipitation followed by qPCR analysis. As shown in Figure 2C, ectopic miR-18a expression significantly increased the amount of the target mRNAs in AGO2 immunocomplexes. In contrast, AGO2 binding of B-cell translocation gene 2 (*BTG2*) and eukaryotic translation initiation factor 4E binding protein 2 (*EIF4EBP2*), which are validated targets of miR-21 [16], was not affected by ectopic expression of miR-18a. Notably, ectopic miR-18a expression slightly increased the cell growth rate *in vitro* (Figure 2D), which is in agreement with a previous report showing that miR-18a overexpression enhances proliferation of K562 and hepatoma cells [24, 25].

**Figure 3:**
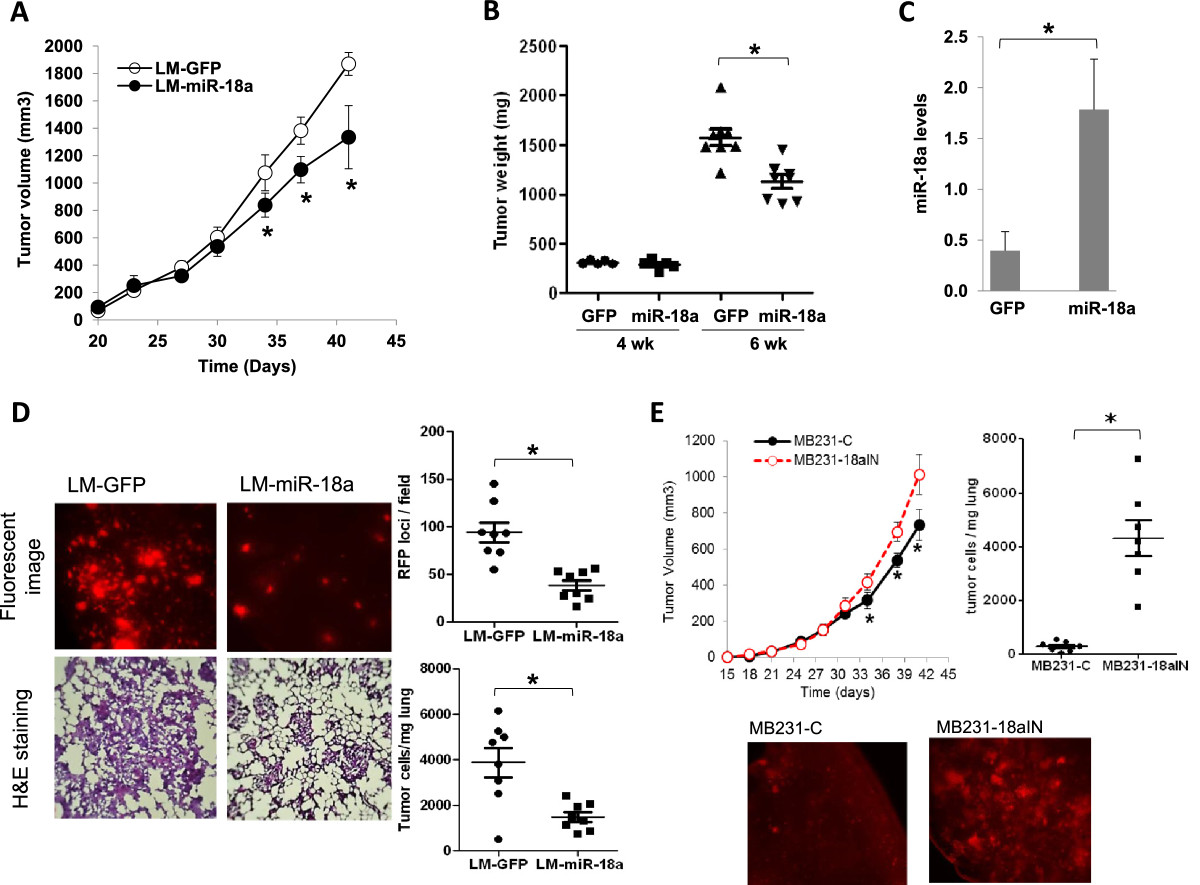
**Effects of ectopic microRNA-18a expression on primary tumor growth and spontaneous lung metastasis. (A)** Growth curves of primary tumors generated from lung metastatic and green fluorescent protein microRNA-18a cells (LM-miR-18a and LM-GFP, respectively). The results are presented as mean ± SE (*n* = 10). *P < 0.05 by Kruskal-Wallis analysis of variance followed by Dunn’s multiple-comparisons test. **(B)** Wet weights of primary tumors that were surgically removed at either 4 weeks or 6 weeks postinoculation. *P < 0.05 by Kruskal-Wallis analysis of variance followed by Dunn’s multiple-comparisons test. **(C)** Expression levels of miR-18a in end-stage primary tumors. Total RNA was prepared from primary tumor tissues harvested 6 weeks after inoculation and subjected to quantitative PCR (qPCR) analysis for miRNA expression. The results are presented as mean ± SD (*n* = 3). *P < 0.05 by Student’s *t*-test. **(D)** Spontaneous lung metastasis from orthotopic sites. Metastatic foci of red fluorescent protein (RFP)–expressing cells on the dorsal surface of the left lung lobe were imaged and counted using a fluorescence microscope at × 10 magnification. *P < 0.05 by Mann-Whitney *U* test (*n* = 8). The presence of tumor cells in the lungs was visualized by hematoxylin and eosin (H&E) staining of formalin-fixed lung sections (10 μM) and quantified by qPCR using the human-specific primers of Alu repeats (hAlu-qPCR). *P < 0.05 by Mann-Whitney *U* test (*n* = 8). **(E)** Growth curves of primary tumors and images of lung metastasis generated from MDA-MB-231 sublines with low miR-18a activity (MB231-18aIN) and control cells expressing mCherry only (MB231-C). Tumor volumes are presented as mean ± SE (*n* = 14). Metastatic foci of mCherry-expressing cells on the dorsal surface of the left lung lobe were imaged using a fluorescence microscope at × 10 magnification. The presence of tumor cells in the lungs was quantified by hAlu-qPCR. *P < 0.05 by Mann-Whitney *U* test (*n* = 7). *n*, the number of experimental repeats.

We next examined the effect of ectopic miR-18a expression on primary tumor growth *in vivo*. LM-miR-18a and LM-GFP cells (2.5 × 10^5^) were inoculated into the fourth inguinal mammary gland fat pads of 4-week-old NSG mice. All females developed palpable tumors within 2 weeks. Both the LM-miR-18a and LM-GFP tumors exhibited similar growth rates until grown to a volume of approximately 500 mm^3^ (Figure 3A and 3B). At this point of tumor progression (day 35 posttransplant), we observed slower growth of the tumors derived from LM-miR-18a cells. The differences in tumor growth were confirmed by comparing end-stage tumor wet weight (6 weeks after inoculation; Figure 3B). Sustained expression of miR-18a in end-stage LM-miR-18a tumors was confirmed by qPCR (Figure 3C). These results suggest that ectopic miR-18a expression does not impact tumor initiation, but instead limits continuous expansion of the tumor mass.

To examine the effect of ectopic miR-18a expression on spontaneous lung metastasis, primary tumors were surgically removed at 4 weeks after inoculation, when the tumor sizes were equivalent (LM-GFP: 235 ± 75 mm^3^ vs LM-miR-18a: 231 ± 84 mm^3^). Lung metastases were evaluated 5 weeks after primary tumor resection via gross examination of the dorsal surface of the left lung lobes under a fluorescence microscope, followed by examination of H&E-stained tissue sections. The number of lung metastatic foci in mice inoculated with LM-miR-18a cells was significantly reduced compared to that of mice inoculated with LM-GFP cells (Figure 3D). Likewise, the number of tumor cells detected by qPCR using primers specific for the human Alu sequence in the lungs of mice inoculated with LM-miR-18a was reduced by about 60% compared to lungs of mice inoculated with LM-GFP (3,877 ± 641.6 cells/mg vs 1,496.0 ± 208.8 cells/mg lung tissue, respectively (*n* = 8) (Figure 3D). When inspecting tissue sections (10 μm thick) from mice inoculated with LM-miR-18a cells, we observed similar intensities of GFP (coexpressed with miR-18a) in lung metastatic cells and primary tumor cells, suggesting that the ectopically overexpressed miR-18a was retained in lung metastases. Taken together, these results demonstrate that ectopic miR-18a expression reduced spontaneous lung metastasis from the MFPs.

We next examined the effect of inhibiting miR-18a expression on spontaneous lung metastasis in MDA-MB-231 cells. A lentiviral vector expressing miArrest inhibitor of miR-18a and mCherry (HmiR-AN0255-AM03) was used to establish the subline MB231-18aIN. In contrast to ectopic miR-18a expression, which reduced late-stage tumor growth and lung metastasis, miR-18a inhibition enhanced late-stage tumor growth and substantially increased spontaneous lung metastasis (Figure 3E). Specifically, miR-18a inhibition increased final tumor wet weight by about 20% (MB231-18aIN: 1,012 ± 278 mg (*n* = 14) vs MB231-C: 852 ± 230 mg (*n* = 14), *P* = 0.0743 by unpaired *t*-test with Welch’s correction, 6 weeks posttransplant) and increased cell numbers in lung tissues more than 10-flold as measured by hAlu-qPCR (MB231-18aIN: 4,274 ± 1,978 cells/mg lung tissue (*n* = 7) vs MB231-C: 294 ± 183 cells/mg lung tissue (*n* = 7); *P* < 0.001 by unpaired *t*-test with Welch’s correction).

### miR-18a regulates HIF1α activity and cell response to hypoxia

HIF1A activation by hypoxia has been identified as a key event for continuous expansion of tumor mass and metastasis by stimulating angiogenesis and activating the expression of metastatic signature genes in various types of tumor cells [18, 26–28]. We identified an evolutionarily conserved miR-18a target site located in the 3′-UTR of *HIF1A* mRNA, consistent with observations that ectopic miR-18a expression significantly increased *HIF1A* mRNA interaction with RISC (Figure 2B). Therefore, we hypothesized that miR-18a might affect tumor growth and metastasis by targeting HIF1A.

**Figure 4:**
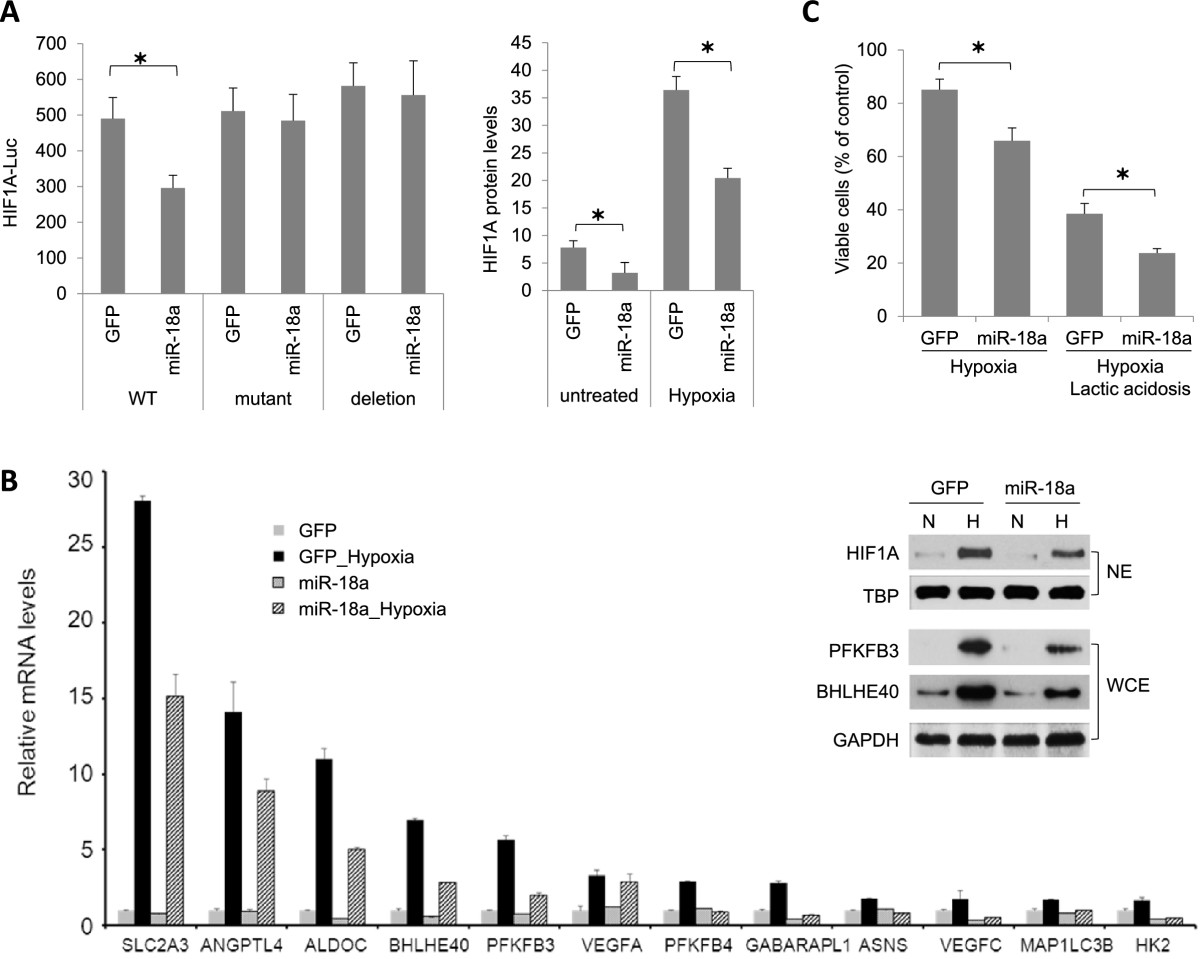
**Effects of microRNA-18a on hypoxia-inducible factor 1α expression and hypoxic response. (A)** MicroRNA-18a (miR-18a) directly targeted the 3′ untranslated region (3′-UTR) of hypoxia-inducible factor 1α (*HIF1A*) mRNA and ectopic miR-18a expression decreased HIF1A protein levels in MB231RN-LM cells cultured at either normoxia or hypoxia (2% O_2_ for 16 hours). HIF1A-3′-UTR-Luciferase reporter activities were measured in MDA-MB-231/DROSHA-shRNA cells cotransfected with either pEZX-MR06 (expressing miR-18a) or control vector pEZX-GFP. GFP, Green fluorescent protein; shRNA, Short-hairpin RNA. Luciferase activities were normalized to internal control pSV-β-galactosidase and are presented as mean ± SD. *P < 0.05 by Student’s *t*-test (*n* = 4). WT, mutant and deletion represent wild type, miR-18a binding site mutation and miR-18a binding site deletion of HIF1A-3′-UTR-Luciferase reporter, respectively. HIF1A protein levels in MB231RN-LM cells transduced with miR-18a or GFP were measured by a cell-based enzyme-linked immunosorbent assay, normalized to cytochrome c and presented as mean ± SD (*n* = 4). *P < 0.05 by Student’s *t*-test. **(B)** miR-18a attenuated the induction of HIF1A target genes by hypoxia in MB231RN-LM cells. Cells were exposed to hypoxia (2% O_2_ for 16 hours). mRNA levels were measured by quantitative PCR and normalized to *ACTB*, and data are presented as mean ± SD (*n* = 3). The inset shows the expression levels of indicated proteins in nuclear extracts (NEs) or whole-cell lysates (WCEs) detected by immunoblotting. TATA-binding protein (TBP) and glyceraldehyde 3-phosphate dehydrogenase (GAPDH) were used as loading controls for NEs and WCEs, respectively. **(C)** Ectopic miR-18a expression in MB231RN-LM cells reduced viable cell numbers after cells were exposure to hypoxia (2% O_2_) and lactic acidosis (25 mM lactic acid, pH 6.7) for 48 hours. *P < 0.05 by Student’s *t*-test. *n*, the number of experimental repeats.

First, we examined whether miR-18a targets *HIF1A* mRNA through the conserved binding site located in the 3′-UTR using a MDA-MB-231/DROSHA-shRNA subline that is defective in miRNA synthesis [16] and a HIF1A-3′-UTR-Luc reporter. As shown in Figure 4A (left panel), HIF1A-3′-UTR-Luc activity in cells cotransfected with pEZX-MR06 (expressing miR-18a and GFP) was significantly decreased in comparison to cells cotransfected with a control vector (expressing GFP only). Point mutations or deletion of the miR-18a target site (GCACCUU, positions 409 to 415 of the HIF1A 3′-UTR) abolished the inhibitory effect of miR-18a on reporter luciferase expression.

We next compared HIF1A protein expression in cells cultured under normoxia or hypoxia (2% O_2_ for 16 hours) using a cell-based ELISA that measures total HIF1A protein levels in the context of whole cells (Figure 4B). HIF1A protein levels in LM-GFP cells were increased about sixfold in response to hypoxia. LM-miR-18a cells expressed lower levels of HIF1A protein than LM-GFP cells at either normoxia or hypoxia. In agreement with the reduced HIF1A expression, reduced hypoxia induction of a panel of known hypoxia responsive genes was reduced in LM-miR-18a cells, including *PFKBP3* and *BHLHE40*, which were also confirmed at the protein level by Western blotting (Figure 4B).

To examine the consequence of HIF1A reduction by miR-18a on cell growth under hypoxia, alone or in combination with acidosis, environments that are frequently encountered by cells within solid tumors, we measured viable cell numbers after growing cells under hypoxia (2% O_2_) with or without exposure to a high concentration of lactic acid (25 mM) for 48 hours. Ectopic miR-18a expression significantly decreased viable cell numbers when cells were exposed to hypoxia alone or to hypoxia in combination with lactic acidosis (Figure 4C).

**Figure 5:**
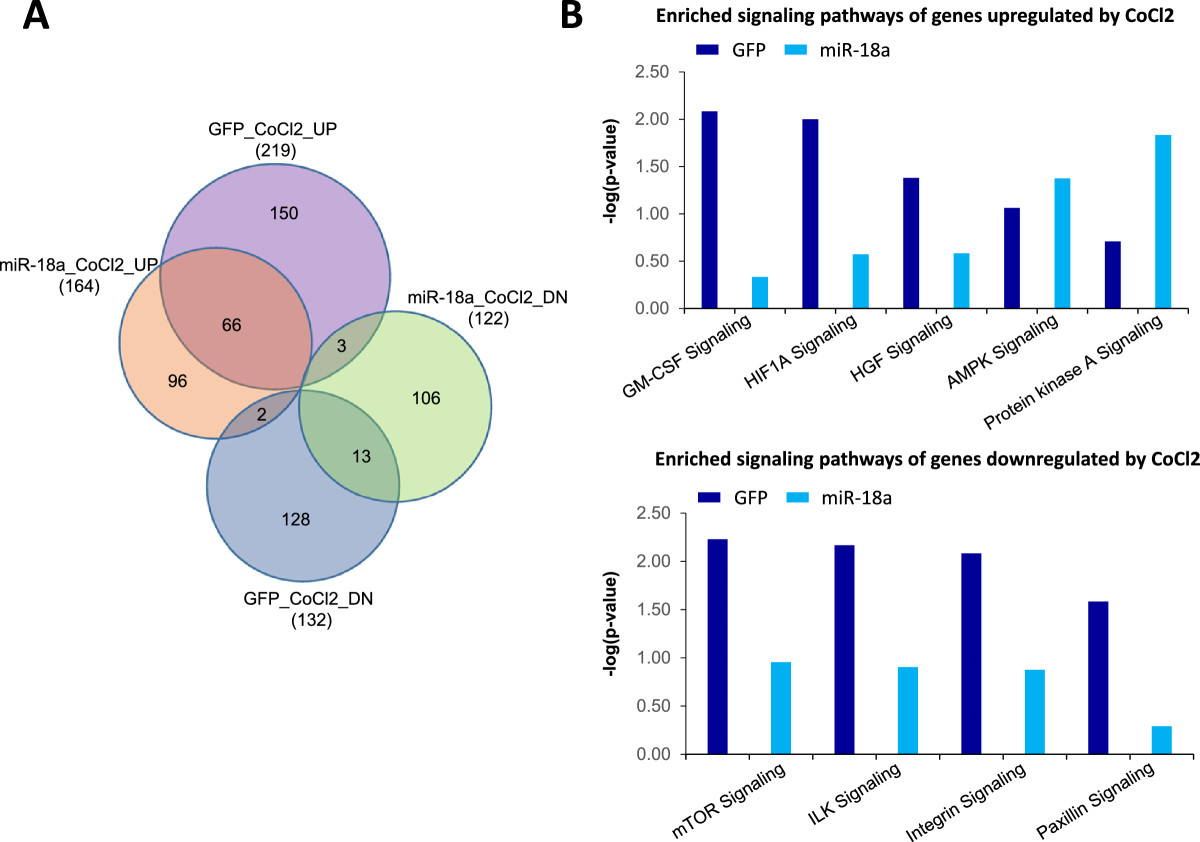
**Ectopic microRNA-18a expression modified gene expression profiles of MB231RN-LM cells in response to CoCl**_**2**_**treatment (200 μM for 4 hours). (A)** Venn diagrams of cobalt(II) chloride (CoCl_2_)–regulated genes (fold change ≥ 1.5, CoCl_2_ () vs untreated, false discovery rate = 0.1) in LM-miR-18a and LM-GFP cells. GFP, Green fluorescent protein; miR-18a, MicroRNA-18a. **(B)** Signaling pathways differentially affected by CoCl_2_ in LM-miR-18a and LM-GFP cells. Enriched signaling pathways of CoCl_2_-regualted genes were identified using Ingenuity Pathway Analysis software. AMPK, 5′ adenosine monophosphate-activated protein kinase; GM-CSF, Granulocyte-macrophage colony-stimulating factor; HGF, Hepatocyte growth factor; HIF1A, Hypoxia-inducible factor 1α; ILK, Integrin-linked kinase; mTOR, Mammalian target of rapamycin.

**Figure 6:**
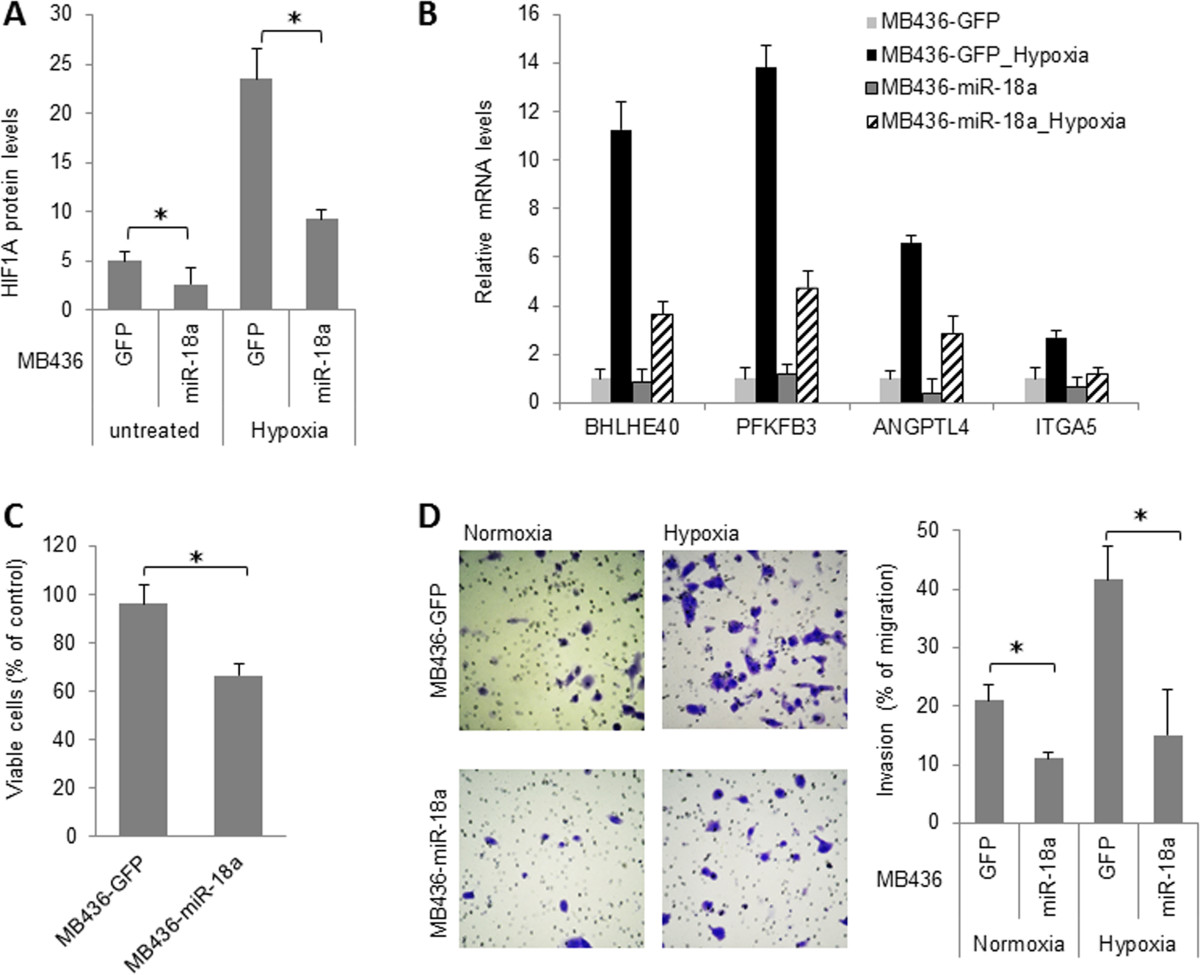
**Effects of microRNA-18a on hypoxia-inducible factor 1α expression and hypoxia response in MDA-MB-436 cells. (A)** Ectopic microRNA-18a (miR-18a) expression decreased hypoxia-inducible factor 1α (HIF1A) protein levels in cells cultured under normoxia or hypoxia (2% O_2_ for 16 hours). HIF1A protein levels were measured using a cell-based enzyme-linked immunosorbent assay and normalized to cytochrome c. The data are presented as mean ± SD (*n* = 4). *P < 0.05 by Student’s *t*-test. GFP, Green fluorescent protein. **(B)** miR-18a attenuated hypoxic gene expression. Cells were exposed to hypoxia (2% O_2_ for 16 hours), and mRNA levels were measured by quantitative PCR and normalized to *ACTB*. Data are presented as mean ± SD (*n* = 3). **(C)** Ectopic miR-18a expression reduced viable cell numbers in cells exposed to hypoxia (2% O_2_ for 72 hours). *P < 0.05 by Student’s *t*-test. **(D)** Ectopic miR-18a expression inhibited cell invasion under normoxia and abolished hypoxia-induced cell invasion. The results are presented as mean ± SD (*n* = 6). *P < 0.05 by Student’s *t*-test. *n*, the number of experimental repeats.

To obtain a more global and unbiased characterization of the effect of miR-18a on HIF1A activity, we compared gene expression profiles of LM-GFP and LM-miR-18a cells treated with CoCl_2_, a hypoxia mimetic that rapidly stabilizes HIF1A protein by inhibiting its prolyl hydroxylation [29, 30]. After exposure to CoCl_2_ (200 μM for 4 hours), 219 or 132 genes were found to be significantly upregulated or downregulated, respectively, in LM-GFP cells (FDR = 0.1, FC > 1.5). All data are reported in the GEO database [GEO:GSE45362]. Consistent with miR-18a-mediated suppression of HIF1A, a smaller number of genes were found to change in response to CoCl_2_ treatment in LM-miR-18a cells, with 164 and 122 genes upregulated or downregulated, respectively (Figure 5A). Ingenuity Pathway Analysis revealed that several signaling pathways were differentially affected by CoCl_2_ in LM-GFP and LM-miR18a cells (Figure 5B). For example, CoCl_2_-induced genes in LM-GFP cells were significantly enriched for HIF1A, granulocyte-macrophage colony-stimulating factor and hepatocyte growth factor signaling pathways, indicating activation of HIF1A and downstream angiogenic signaling pathways. CoCl_2_-downregulated genes in LM-GFP cells were significantly enriched in the integrin, paxillin and integrin-linked kinase signaling pathways involved in cell adhesion and migration. In contrast, in LM-miR-18a cells, none of these known HIF1A regulated pathways were significantly affected by CoCl_2_ exposure. Taken together, these observations provide additional evidence in support of an essential role for miR-18a in regulating HIF1A activity in breast tumors. Notably, according to three target prediction programs (TargetScan, PicTar and miRBase), most of the CoCl_2_-responsive genes affected by miR-18a lack target sites of miR-18a, suggesting that miR-18a regulates CoCl_2_-responsive genes primarily through indirect mechanisms.To investigate whether miR-18a-mediated HIF1A inhibition can be extended to other basal-like breast cancer cell lines, we examined the impact of ectopic miR-18a expression in MDA-MB-436 cells. As shown in Figure 6, miR-18a expression downregulated the expression of HIF1A protein as well as the induction of HIF1A target genes at hypoxia. In addition, ectopic miR-18a expression in MDA-MB-436 cells significantly decreased cell growth under hypoxia (Figure 6C) and abolished hypoxia-induced cell invasion (Figure 6D). Taken together, these observations provide additional evidence that supports a conserved role for miR-18a in limiting HIF1A activity in basal-like breast cancer cells.

### miR-18a represses tumor cell metastatic properties primarily through hypoxia-inducible factor 1α

**Figure 7:**
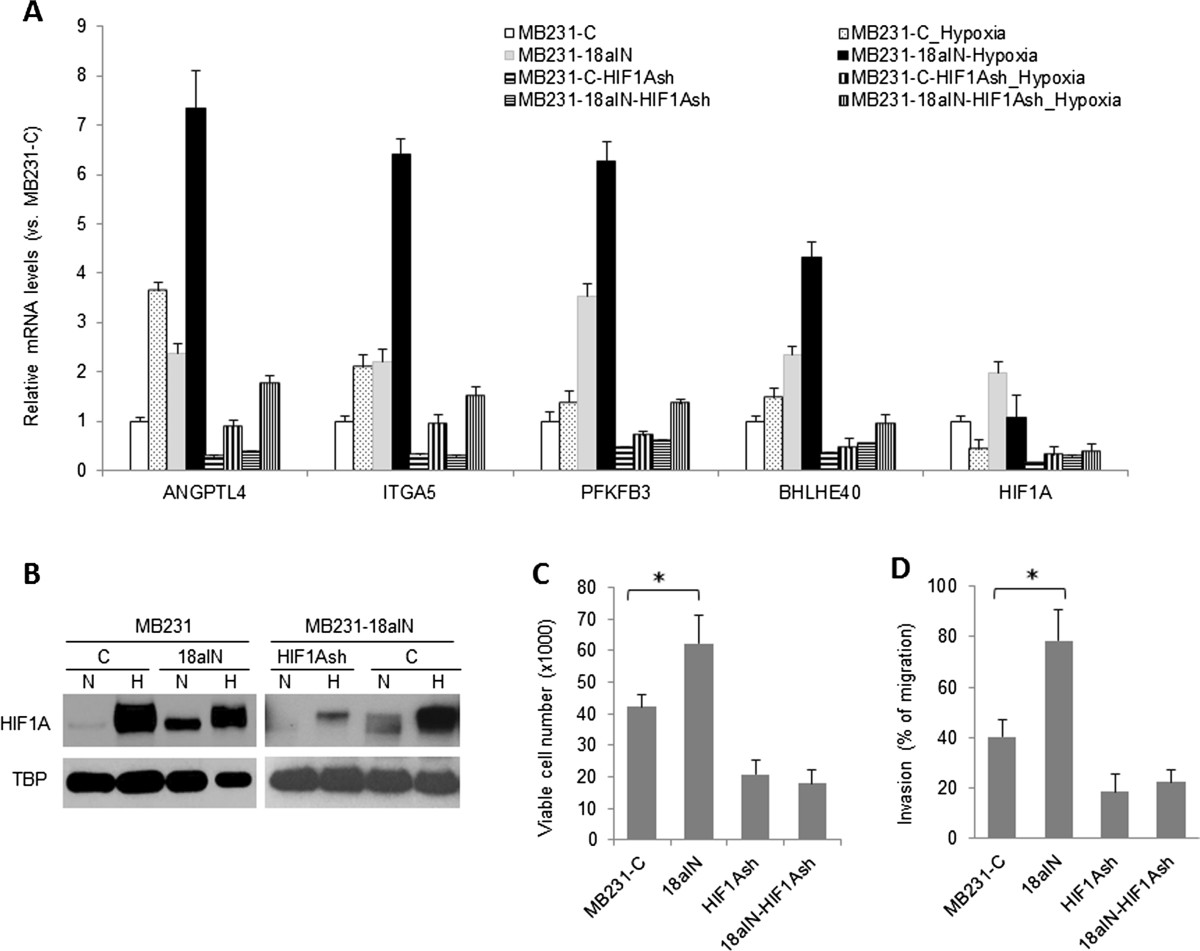
***HIF1A*** **knockdown abolished the effect of microRNA-18a on cell metastatic properties. (A)** Expression fold changes of hypoxia-inducible factor α (*HIF1A*) and hypoxia-responsive genes in MDA-MB-231 cells expressing microRNA-18a (miR-18a) inhibitor and/or HIF1A short-hairpin RNA (shRNA). The mRNA levels were measured by quantitative PCR after cells were exposed to hypoxia (2% O_2_ for 6 hours) and normalized to *ACTB*. Data are presented as mean ± SD (*n* = 3). **(B)** HIF1A protein expression levels in nuclear extracts from untreated or hypoxia-treated (2% O_2_ for 6 hours) cells were detected by immunoblotting assay. TATA-binding protein (TBP) was used as a loading control. **(C)** Effect of miR-18a inhibitor and HIF1A shRNA on viability of cells cultured in suspension for 6 days. **(D)** Effect of miR-18a inhibitor and HIF1A shRNA on cell invasion. The results are presented as mean ± SD (*n* = 4). *P < 0.05 by Student’s *t*-test. *n*, the number of experimental repeats.

In cultured cells, miR-18a inhibition enhanced gene expression induced by hypoxia (2% O_2_ for 6 hours) (Figure 7A). Immunoblotting assay results showed that miR-18a inhibition increased baseline protein levels of HIF1A (Figure 7B, left panel). Consistent with the observation that miR-18a inhibition promoted tumor growth and metastasis *in vivo* (Figure 3E), MB231-18aIN cells were more resistant to anoikis (Figure 7C) and more invasive (Figure 7D) in comparison to control MB231-C cells. To confirm that these cellular changes induced by miR-18a inhibition were mediated by HIF1A, we examined the impact of HIF1A knockdown on the efficacy of a miR-18a inhibitor. Expression of HIF1A shRNA in MB231-C or MB231-18aIN cells reduced *HIF1A* mRNA levels by approximately 70% compared to control cells transduced with empty vector (Figure 7A). The reduced HIF1A expression in MB231-18aIN-HIF1AshRNA cells compared to MB231-18aIN-Vec cells was also confirmed by Western blotting (Figure 7B, right panel). HIF1A knockdown substantially decreased hypoxia-induced gene expression in both MB231-C and MB231-18aIN cells (Figure 7A), including *PFKFB3*, *BHLHE40*, angiopoietin-like 4 (*ANGPTL4*) and integrin α5 (*ITGA5*). Consistent with the known function of HIF1A to protect cells against anoikis and promote cell invasion, HIF1A knockdown substantially reduced the viability of cells cultured in suspension as well as cell invasive activity (Figure 7C and 7D). Importantly, no difference in cell viability was observed in suspension culture or in cell invasion between MB231-C-shHIF1A and MB231-18aIN-shHIF1A cells, demonstrating that the predominant target of miR-18a that mediates phenotypes in MDA-MB-231 cells is HIF1A.

**Figure 8:**
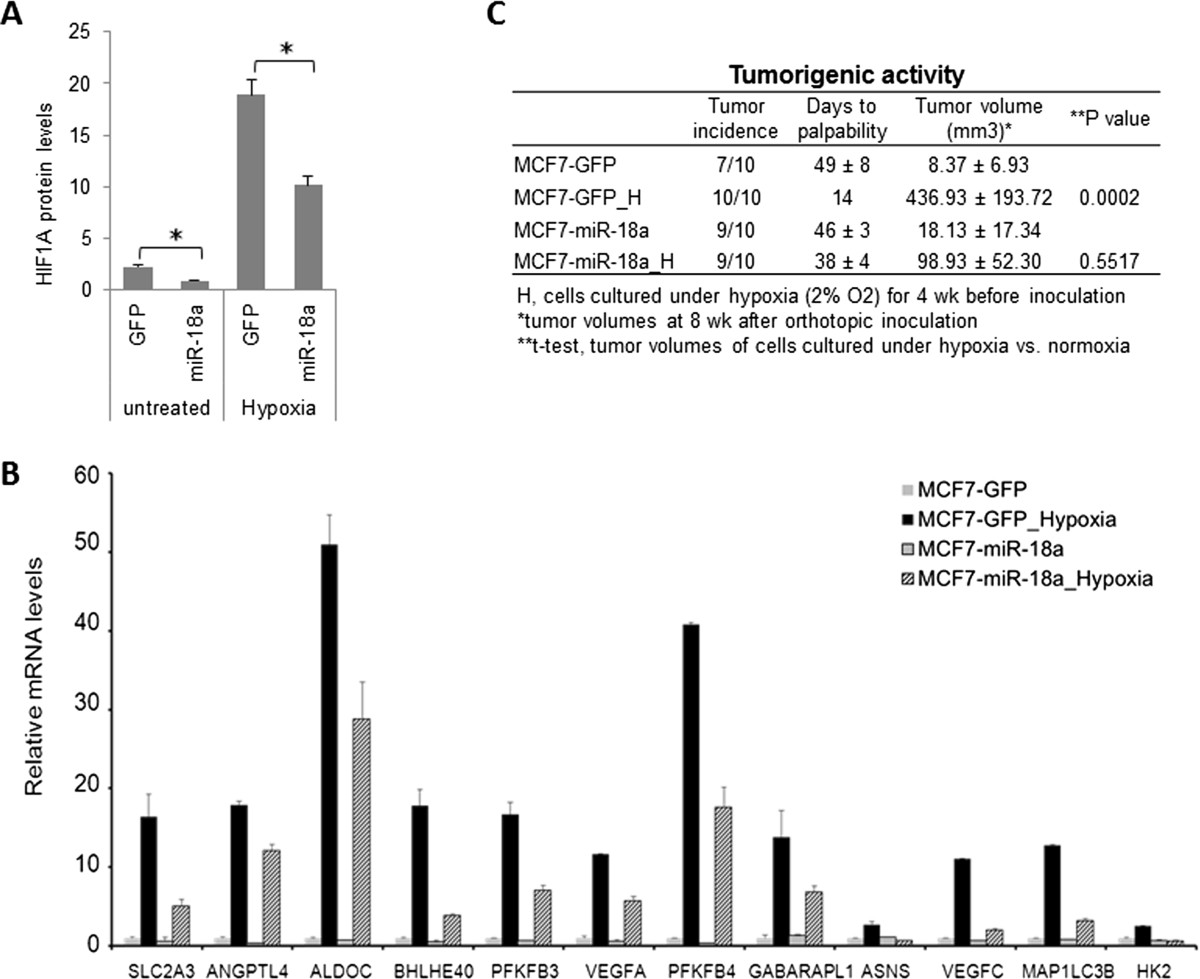
**Ectopic microRNA-18a expression diminished hypoxia induced tumorigenic activity of MCF7 cells. (A)** MicroRNA-18a (miR-18a) expression in MCF7 cells reduced hypoxia-inducible factor 1α (HIF1A) protein expression as measured using a cell-based enzyme-linked immunosorbent assay. GFP, Green fluorescent protein. *P < 0.05 by Student’s t-test. **(B)** miR-18a expression dampened hypoxia-induced gene expression in MCF7 cells. Gene mRNA levels were determined by quantitative PCR and normalized to *ACTB*. **(C)** Exposure to hypoxia (2% O_2_ for 4 weeks) prior to inoculation increased tumorigenic activity of MCF7 cells. The hypoxia-induced tumorigenic activity was abolished by ectopic miR-18a expression.

Ectopic HIF1A expression in MDA-MB-231 cells was previously reported to suppress tumor growth in a xenograft model [29]. Therefore, it is difficult to rescue a miR-18a-mediated suppressive effect on tumor growth and metastasis by ectopic HIF1A expression in LM-miR-18a cells. To provide further evidence that miR-18a regulates HIF1A activity, we examined the effect of ectopic miR-18a-5p (miR-18a) expression on hypoxia-induced tumorigenic activity of MCF7 cells. In an orthotopic xenograft model, MCF7 cells (1 × 10^6^ cells/injection) generated small tumors with a latency period of approximately 7 weeks. Prolonged exposure of MCF7 cells to hypoxia (2% O_2_ for 4 weeks *in vitro*) prior to inoculation substantially reduced the latency period of tumor initiation and enhanced tumor growth. Ectopic miR-18a expression in MCF7 cells reduced HIF1A protein expression (Figure 8A), dampened hypoxia-induced gene expression (Figure 8B) and completely abolished hypoxia-induced tumorigenic activity (Figure 8C). These results provide additional evidence supporting a role for miR-18a in regulating hypoxic response in breast cancer cells.

### Reverse correlation of miR-18a expression with expression of HIF1A and panel of hypoxia-responsive genes in basal-like breast tumors

**Figure 9:**
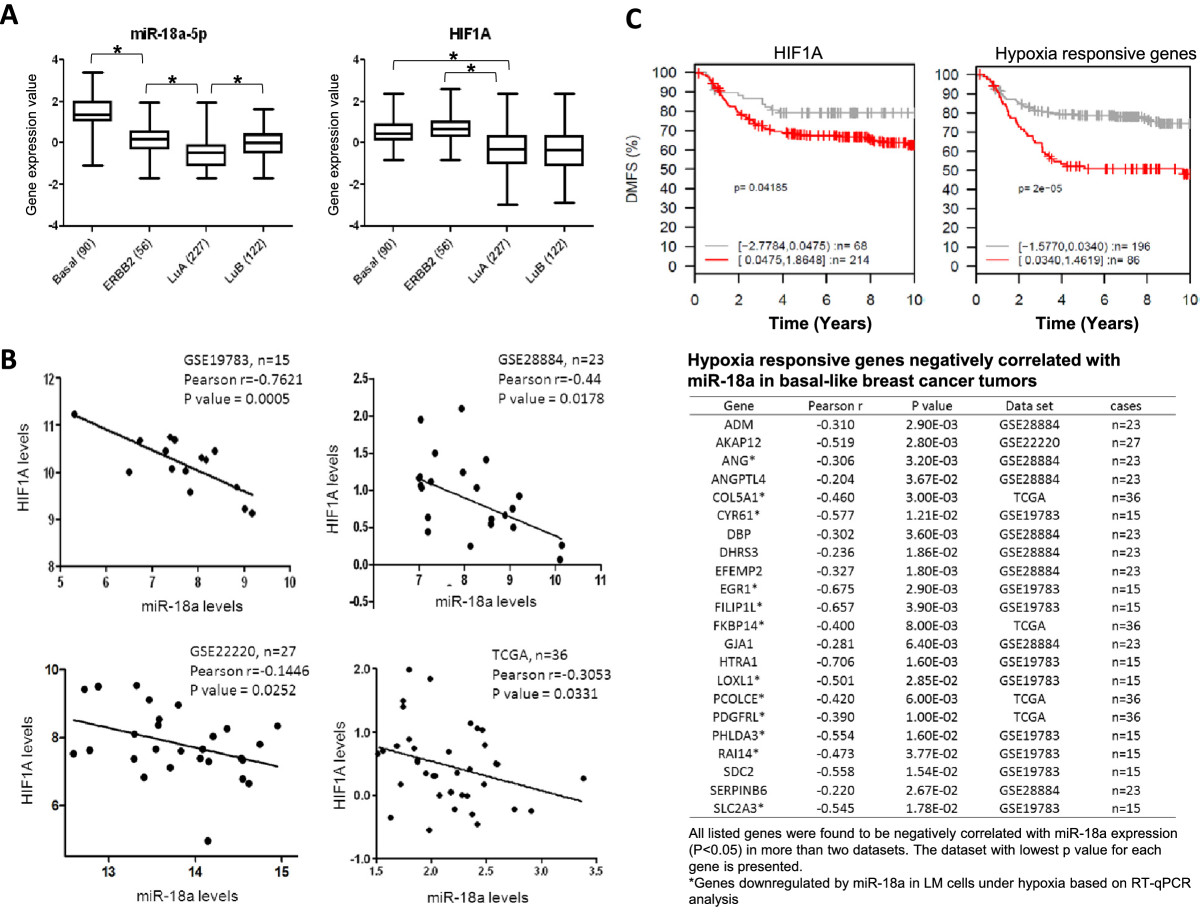
**Correlation of microRNA-18a expression with** ***HIF1A*** **mRNA expression, hypoxia-responsive gene expression and distant metastasis in breast tumors. (A)** Differential expression of microRNA-18a (miR-18a) and hypoxia-inducible factor 1α (*HIF1A*) mRNA in breast cancer subtypes. Expression data were retrieved from the Cancer Genome Atlas database. GE, Generalized estimating equation. **(B)** There was an inverse correlation between miR-18a and *HIF1A* mRNA expression in basal-like breast tumors. The basal-like breast tumors in four individual data sets were defined according to the PAM50 classifier. **(C)** Higher expression of *HIF1A* and hypoxia-responsive genes is associated with a shorter interval of distant metastasis–free survival (DMFS) of patients with basal-like breast tumors. Kaplan-Meier analysis was conducted using the GOBO program.

To examine the clinical relevance of miR-18a mediated HIF1A inhibition, we investigated the relationship between miR-18a and *HIF1A* mRNA levels in human breast tumor specimens by analyzing four data sets that contain both mRNA and miRNA expression data, which are available from TCGA or in the GEO database [GEO:GSE19783, GEO:GSE22220, GEO:GSE28884] [30–33]. The expression of miR-18a, along with other *MIR17HG* family members, was higher in basal-like tumors than in the other molecular subtypes defined by the PAM50 classifier [19] (Figure 9A, left panel). *HIF1A* mRNA is highly expressed in both basal and ERBB2 subtypes compared to luminal subtypes (Figure 9A, right panel), in agreement with the observation that HIF1A protein is expressed at higher levels in basal cancers relative to luminal cancers [34]. Within the basal-like breast tumors, we found a significant inverse correlation between the expression levels of miR-18a and *HIF1A* mRNA in all four data sets (Figure 9B). This correlation was not detected for any other subtype of breast cancer. These results implicate a role for miR-18a in restraining *HIF1A* activity in basal-like breast tumors.

We further examined the correlation of the expression levels of miR-18a and hypoxia-responsive genes that were associated with distant metastasis of breast tumors. The hypoxia-responsive genes in basal-like breast cancer cells were retrieved from GEO data sets [GEO:GSE17188, GEO:GSE18494] [28, 35]. The correlation of hypoxia-responsive gene expression with DMFS interval was assessed using the GOBO algorithm, which analyzes the correlation of gene expression and clinicopathological characteristics using expression data of 1,881 breast tumor specimens annotated with clinical information [33]. A panel of hypoxia-responsive genes was found to be associated with reduced DMFS interval of patients with basal-like tumors (*P* < 0.05 by Kaplan-Meier analysis with 10-year censoring). We examined their correlation with miR-18a expression in the above-mentioned four data sets, which contain both mRNA and miRNA expression data. This analysis led to the identification of a panel of metastasis-associated hypoxic genes that negatively correlated with miR-18a (Figure 9C). The individual or collective roles of these genes in mediating the miR-18a-dependent suppression of lung metastasis in basal breast cancers warrant further studies.

## Discussion

Deregulation of *MIR17HG* has been linked to breast cancer progression, but the function of each individual miRNA encoded by *MIR17HG* in breast tumor cells has not yet been fully defined [9, 12, 36–38]. Compared to other family members of *MIR17HG*, miR-18a is less studied, and only limited information is available regarding its function in human cancer cells. Regarding luminal breast cancer cells, miR-18 has been shown to inhibit cell proliferation by targeting ESR1 [11]. In glioblastomas, miR-18a has been reported to repress tumor progression through targeting SMAD3 and CTGF [39]. Here we report evidence that miR-18a plays an important role in restricting tumor growth and pulmonary metastasis of basal-like breast cancer by regulating HIF1A expression and hypoxic response. HIF1A was reported as a target of *MIR17HG* in lung cancer cells, but the specific role of miR-18a in the control of HIF1A expression and activity was not tested [40].

We found that miR-18a, along with other family members encoded by *MIR17HG*, was downregulated in breast cancer cells that spontaneously metastasized to lungs in an orthotopic xenograft model. We further confirmed that *MIR17HG* downregulation is an acquired trait associated with spontaneous lung metastasis by using orthotopic xenograft models derived from cell colony expansion. This finding is in line with the previous observation that *MIR17HG* expression is downregulated in human primary breast tumors with positive lymph node metastasis compared to those with no metastasis [12]. *MIR17HG* downregulation during metastatic progression is also congruent with the reported inverse correlation between expression of miR-92a, another member encoded by *MIR17HG*, and the tumor grade and recurrence-free survival of breast cancer patients [38]. However, loss of *MIR17HG* expression was not recognized in a previous study on lung metastatic derivatives of MDA-MB-231 cells [41]. The discrepancy could be caused by differences in experimental approach, including the use of different mouse strains and different inoculation routes of tumor cells than those we employed in our present study.

The results of our *in vivo* studies with orthotopic xenograft models of the metastatic MDA-MB-231 variant demonstrate that ectopic miR-18a expression significantly attenuated continuous expansion of primary tumor mass and decreased spontaneous lung metastasis. In contrast, miR-18a inhibition increased primary tumor growth and lung metastasis. These observations support a role of miR-18a in repressing tumor mass expansion and distant metastasis. Cells in solid tumors commonly encounter fluctuations in nutrient and oxygen availability caused by unbalanced cell proliferation and blood vessel development [26]. Continuous tumor growth is dependent on adaptation of tumor cells to hypoxic stresses and angiogenesis, two processes that are primarily regulated by HIF1A [42]. In addition, mounting evidence suggests a critical role of hypoxia in promoting tumor metastatic progression by rendering cells resistant to apoptosis and invasive features. The results of our mechanistic studies provide evidence that miR-18a directly regulates HIF1A expression and consequently the expression of hypoxia-responsive genes and cell response to hypoxia.

Expression data analysis using a metadata set composed of data from 1,881 breast tumors [20] revealed that higher expression of *HIF1A* and a panel of hypoxia-responsive genes is associated with shorter DMFS interval of patients with basal-like tumors, but not other subtypes. This finding is in agreement with previous reports that enhanced hypoxic response is associated with poor prognosis and chemotherapy resistance of basal-like tumors [43, 44] and supports the notion that HIF1A activation drives metastatic progression of basal-like tumors. Importantly, on the basis of four independent data sets containing both miRNA and mRNA expression data, we found that miR-18a expression is inversely correlated with the expression of *HIF1A* and hypoxia-responsive genes in basal-like tumors. However, the available samples of basal-like tumors with both miRNA expression and DMFS data are not sufficient to establish the prognostic value of miR-18a for distant metastasis. Taken together, our observations suggest a role of miR-18a in suppressing metastasis by restricting hypoxic response in basal-like breast tumors.

Basal-like tumors are characterized by high proliferation rates and rapid mass expansion, which lead to cyclic hypoxia and reoxygenation. Fine-tuned HIF1A activity by miR-18a may represent an essential mechanism to balance cell proliferation, survival and metastasis. In general, activation of HIF1A and hypoxia-responsive genes puts tumor cells in a survival and invasive state with reduced proliferation [45–48]. High expression of miR-18a in basal-like tumors (Figure 9A) may play a role in promoting cell growth when oxygen supply becomes abundant by restricting the antiproliferative activity of the HIF1A signaling pathway. This notion is consistent with previously report findings that tumors derived from transformed *HIF1A*^−/−^ astrocytes grew faster than its *HIF1A*^*+/+*^ counterpart when placed in the vascular-rich brain parenchyma [49]. However, cells under hypoxic stress rely on enhanced HIF1A activation to survive and obtain invasive features. Constitutive high miR-18a expression might be detrimental to tumor mass expansion and metastatic progression by restraining hypoxic response and consequently deterring hypoxia-induced apoptotic resistance and invasion.

The outcome of hypoxia-induced HIF1A activation was shown to be cell context– and tumor microenvironment–dependent [47, 49]. As an upstream regulator of HIF1A, miR-18a function is likely tumor type–dependent. This notion is in line with previously reported findings that miRNAs encoded by *MIR17HG* promote development of B-cell lymphomas and lung cancer, but repress glioblastoma progression [38]. On the basis of our study results, we cannot exclude the possibility that other targets may contribute to miR-18a-mediated suppression on tumor growth and metastasis. By integrated analysis of genes regulated by miR-18a under normoxia using a data set from this study [GEO:GSE45362], miRNA target sites, expression correlation of mRNA and miRNA, and correlation with DMFS of patients with basal-like breast tumors, we discovered that miR-18a potentially targets the prometastatic gene matrix metallopeptidase 3 (*MMP3*). Since *MMP3* is a hypoxia-responsive gene, miR-18a may modify the outcome of hypoxia by modulating the expression of specific hypoxia-responsive genes in addition to a general attenuation of hypoxic response by directly targeting HIF1A. It remains to be determined whether MMP3 downregulation contributes to miR-18a-mediated metastasis suppression.

## Conclusions

The results of this study reveal a novel role for miR-18a in repressing metastasis of basal-like breast tumors by restricting HIF1A activity and regulating cell responses to hypoxia. The inverse correlation between miR-18a and *HIF1A* and hypoxia signature genes in basal-like breast tumors is significant in clinical samples and is consistent across multiple data sets. However, understanding how fluctuating miR-18a and HIF1A levels ultimately drive metastasis from the primary tumor to reduce survival requires further investigation.

## Authors’ original submitted files for images

Below are the links to the authors’ original submitted files for images. Authors’ original file for figure 1 Authors’ original file for figure 2 Authors’ original file for figure 3 Authors’ original file for figure 4 Authors’ original file for figure 5 Authors’ original file for figure 6 Authors’ original file for figure 7 Authors’ original file for figure 8 Authors’ original file for figure 9

## Abbreviations

CoCl_2_: Cobalt(II) chloride
DMFS: Distant metastasis-free survival
GFP: Green fluorescent protein
HIF1A: Hypoxia-inducible factor 1α
microRNA-18a: MiR-18a
miRNA: MicroRNA
RFP: Red fluorescent protein
RISC: RNA-induced silence complex.
