# Supplementary figures and images for: MicroRNA-18a inhibits hypoxia-inducible factor 1α activity and lung metastasis in basal breast cancers
Source: Breast Cancer Res. 2014 Jul 28;16:R78. doi: 10.1186/bcr3693 (PMC4405876; doi:10.1186/bcr3693)

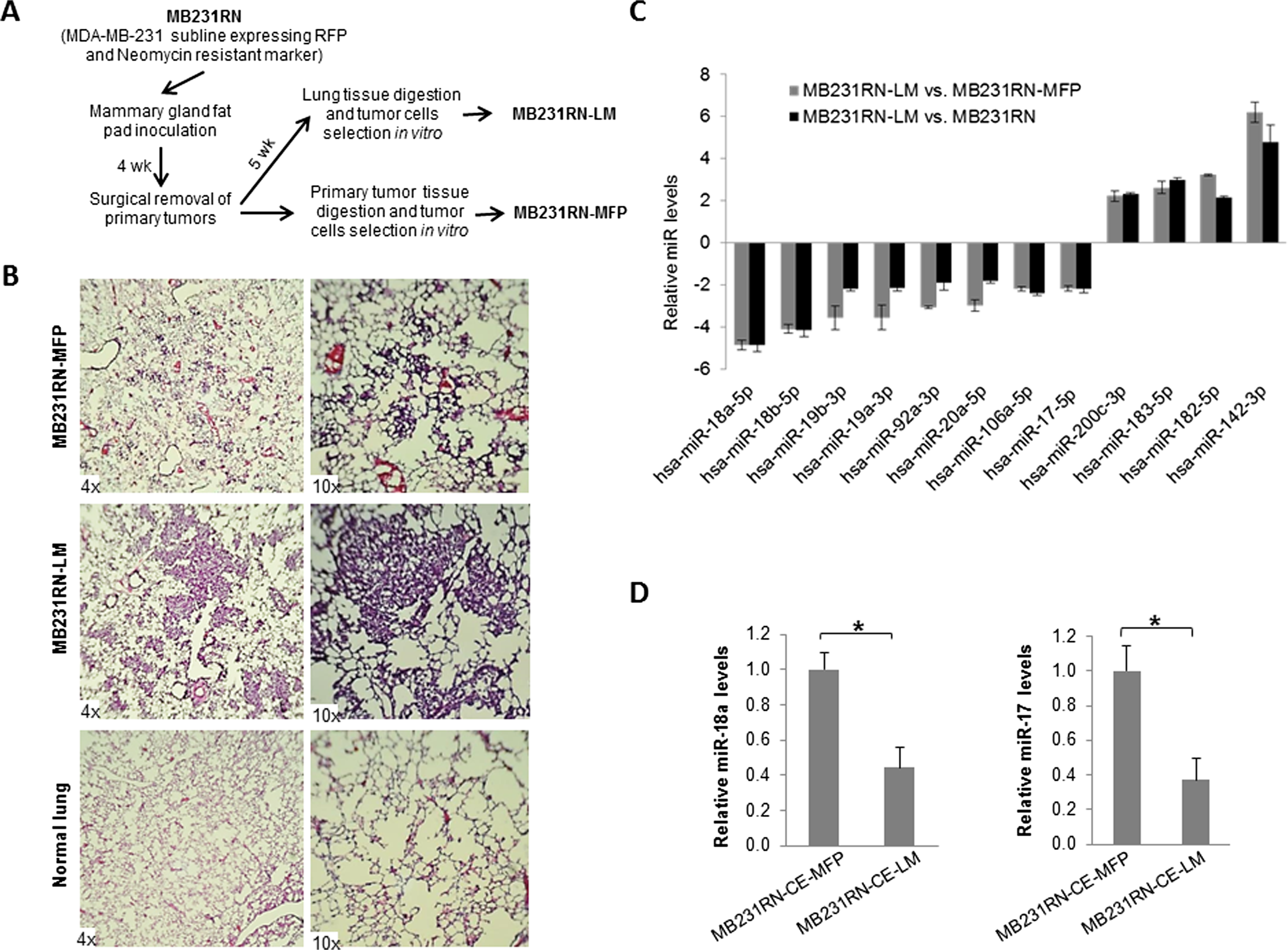

Supplement: Supplementary file 1 — Authors’ original file for figure 1 [file 13058_2013_3438_MOESM1_ESM.tif]

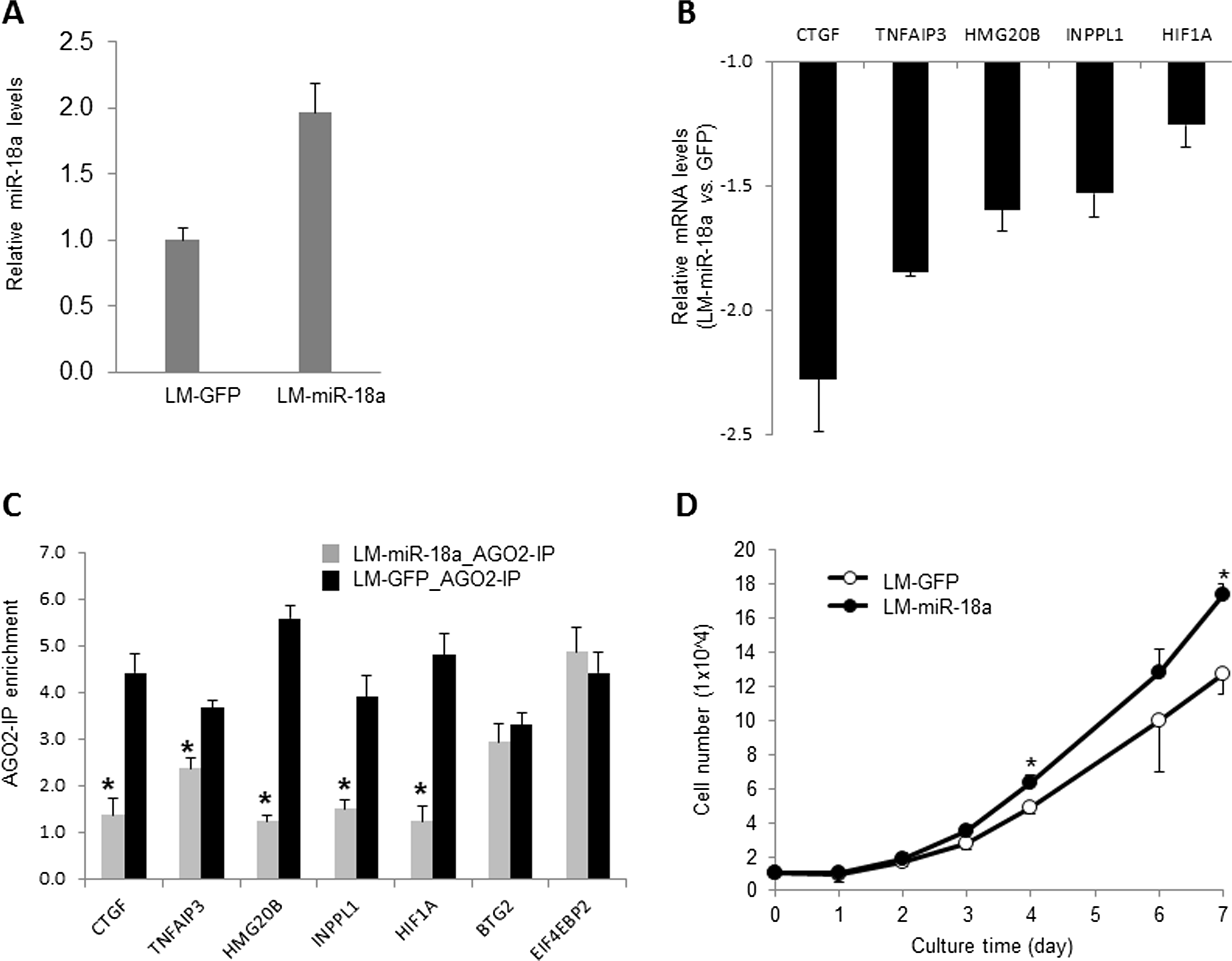

Supplement: Supplementary file 2 — Authors’ original file for figure 2 [file 13058_2013_3438_MOESM2_ESM.tif]

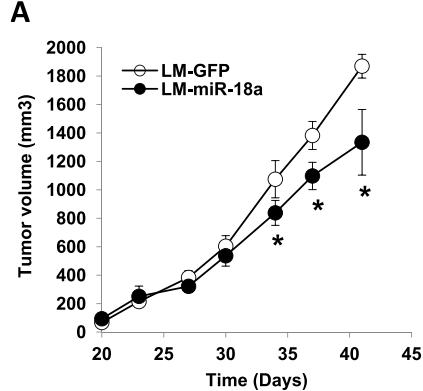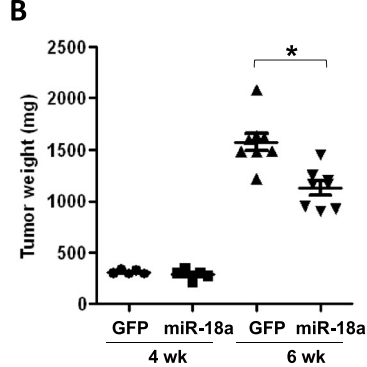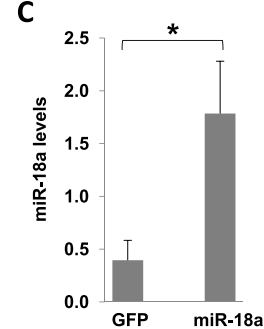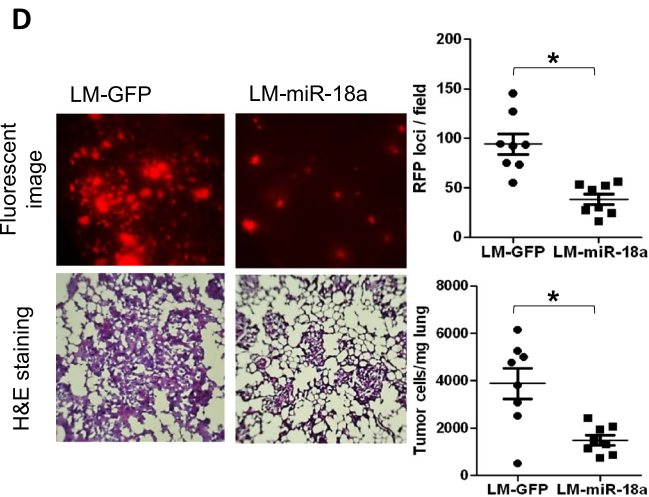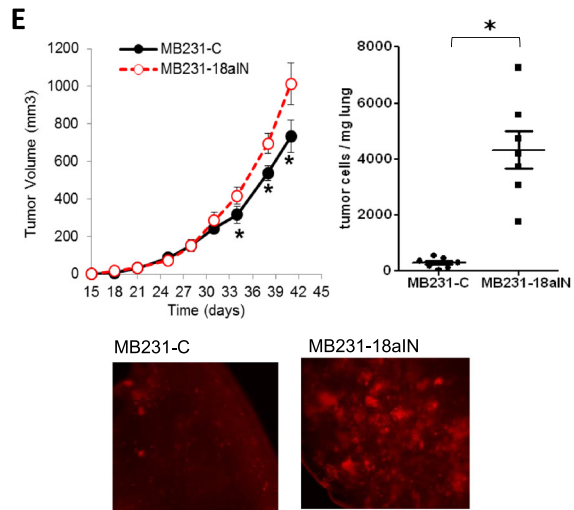

Supplement: Supplementary file 3 — Authors’ original file for figure 3 [file 13058_2013_3438_MOESM3_ESM.pdf]

**A**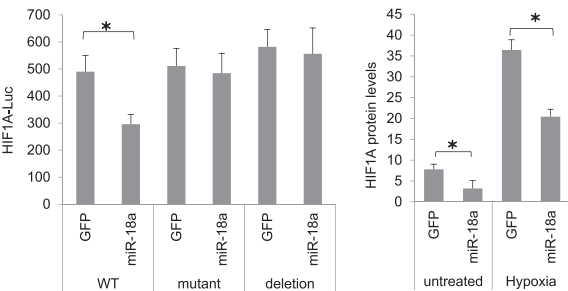**C**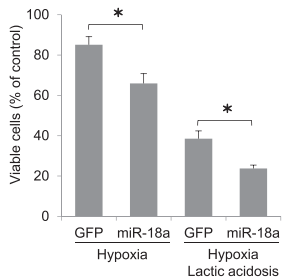**B**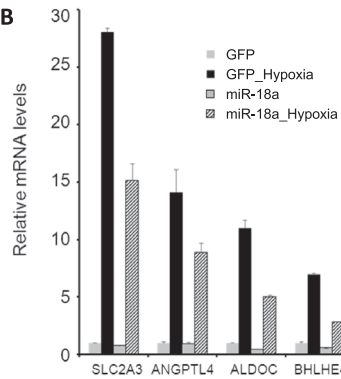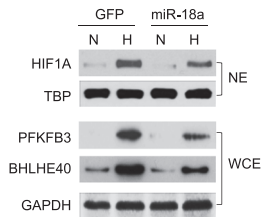

Supplement: Supplementary file 4 — Authors’ original file for figure 4 [file 13058_2013_3438_MOESM4_ESM.pdf]

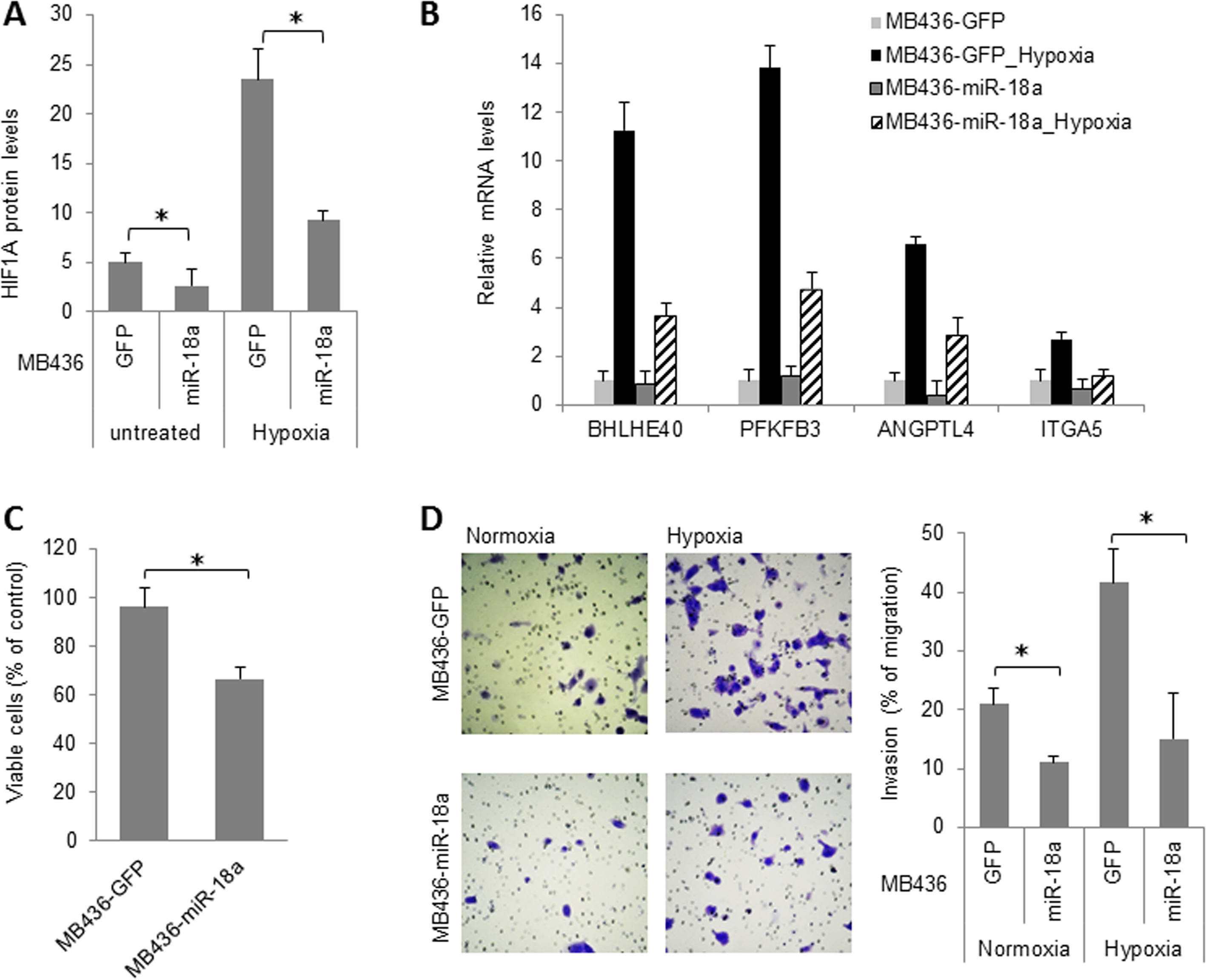

Supplement: Supplementary file 6 — Authors’ original file for figure 6 [file 13058_2013_3438_MOESM6_ESM.tif]

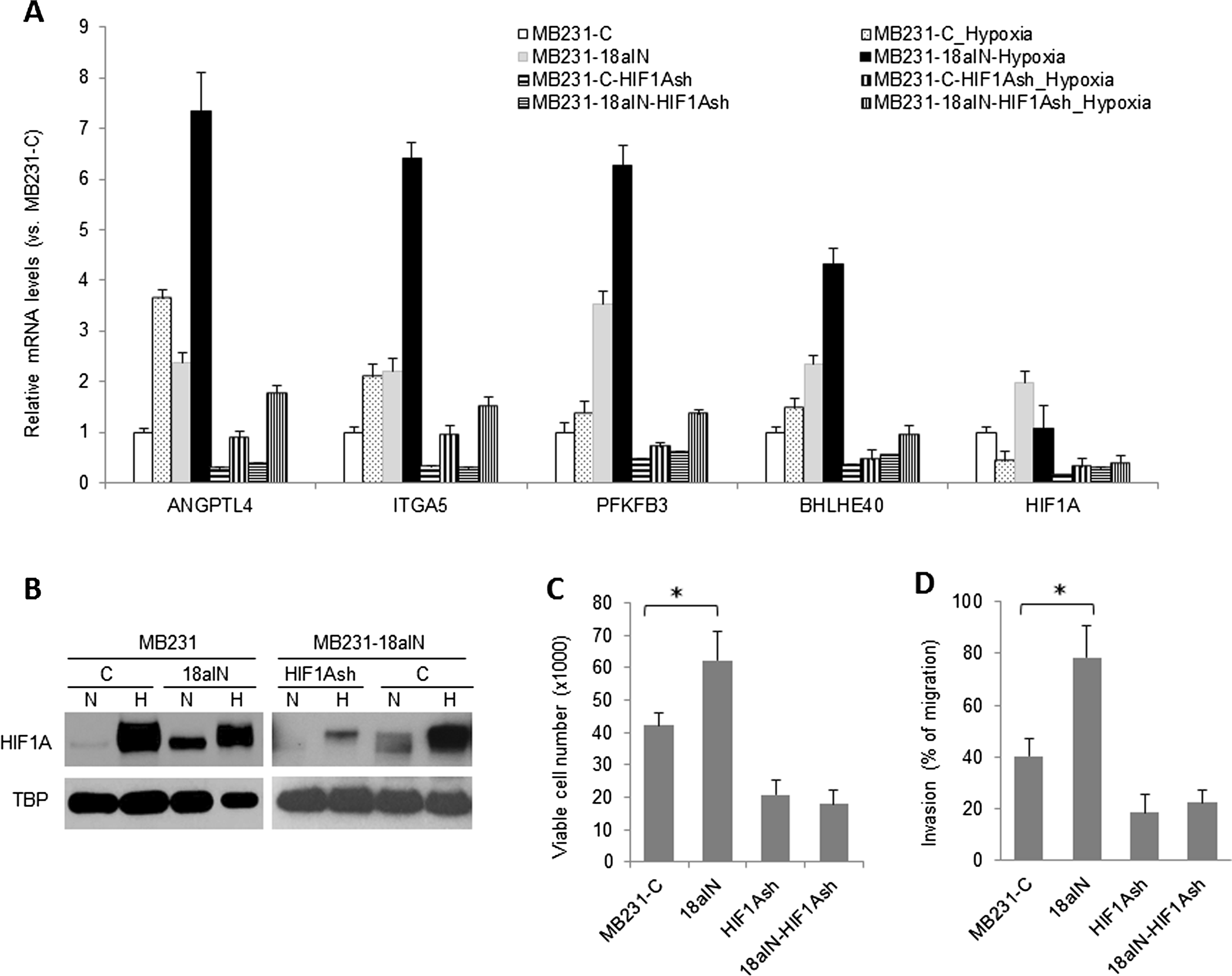

Supplement: Supplementary file 7 — Authors’ original file for figure 7 [file 13058_2013_3438_MOESM7_ESM.tif]

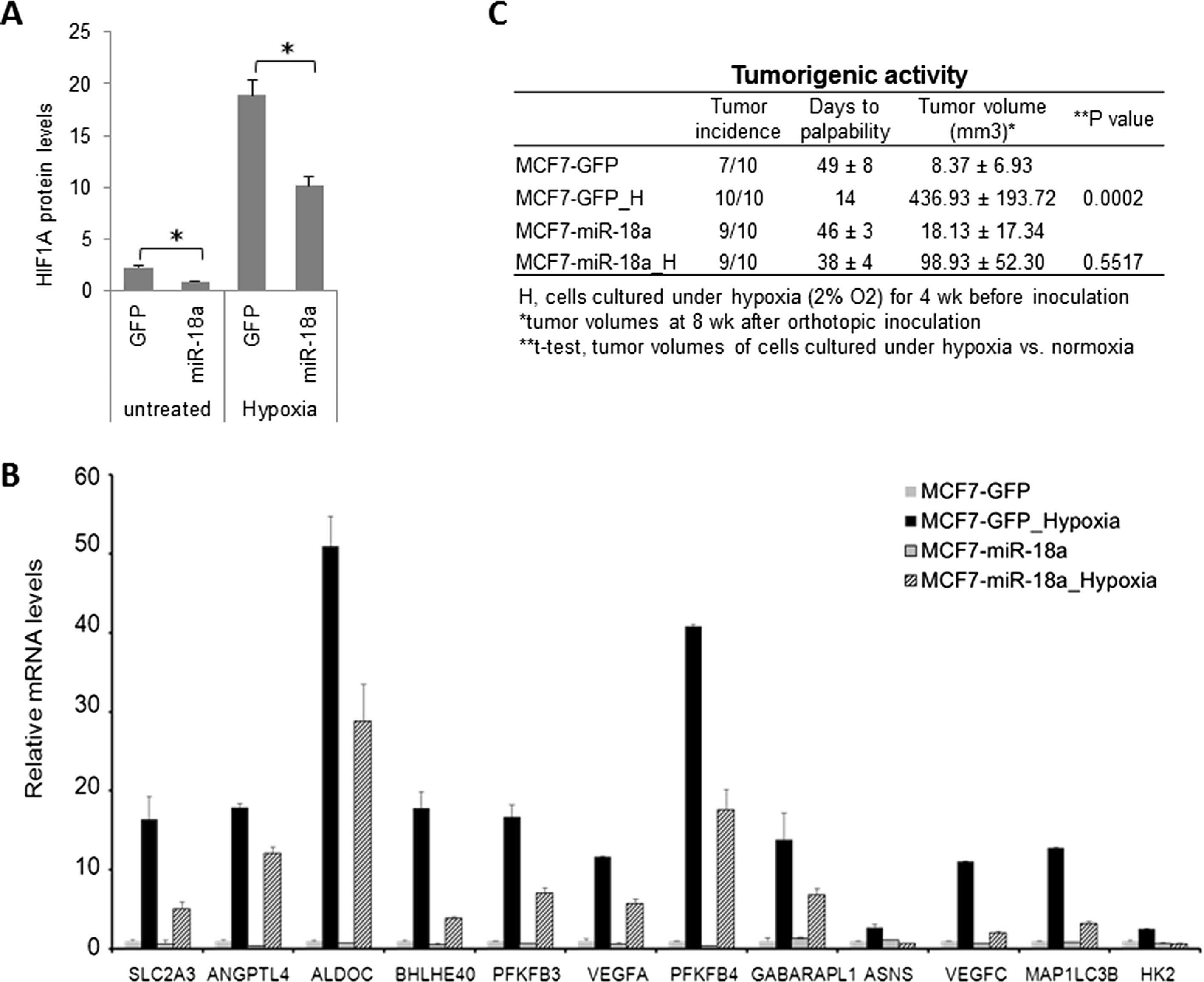

Supplement: Supplementary file 8 — Authors’ original file for figure 8 [file 13058_2013_3438_MOESM8_ESM.tif]
