# Supplementary material for: MicroRNA-18a inhibits hypoxia-inducible factor 1α activity and lung metastasis in basal breast cancers
Source: Breast Cancer Res. 2014 Jul 28;16:R78. doi: 10.1186/bcr3693 (PMC4405876; doi:10.1186/bcr3693)
Supplement: Supplementary file 5 — Authors’ original file for figure 5 [file 13058_2013_3438_MOESM5_ESM.pdf]

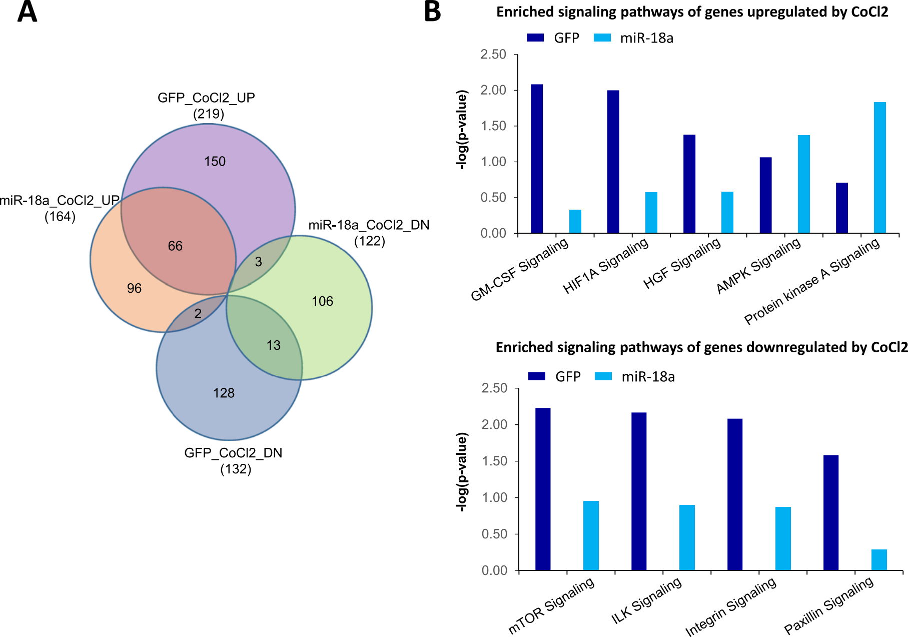
A
GFP_CoCl2_UP
(219)
150
miR-18a_CoCl2_UP
(164)
66
96
3
2
miR-18a_CoCl2_DN
(122)
106
13
128
GFP_CoCl2_DN
(132)
B
Enriched signaling pathways of genes upregulated by CoCl2
GFP
miR-18a
-log(p-value)
2.50
2.00
1.50
1.00
0.50
0.00
GM-CSF Signaling
HIF1A Signaling
HGF Signaling
AMPK Signaling
Protein kinase A Signaling
Enriched signaling pathways of genes downregulated by CoCl2
GFP
miR-18a
-log(p-value)
2.50
2.00
1.50
1.00
0.50
0.00
mTOR Signaling
ILK Signaling
Integrin Signaling
Paxillin Signaling
