# Supplementary material for: MicroRNA-18a inhibits hypoxia-inducible factor 1α activity and lung metastasis in basal breast cancers
Source: Breast Cancer Res. 2014 Jul 28;16:R78. doi: 10.1186/bcr3693 (PMC4405876; doi:10.1186/bcr3693)
Supplement: Supplementary file 9 — Authors’ original file for figure 9 [file 13058_2013_3438_MOESM9_ESM.pdf]

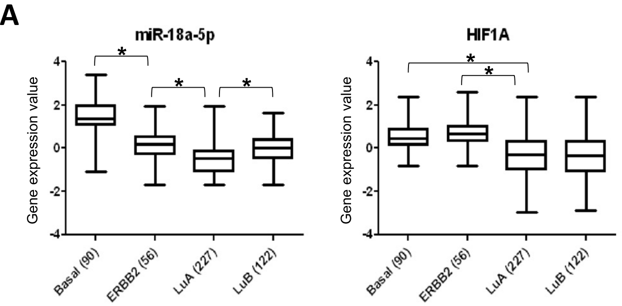

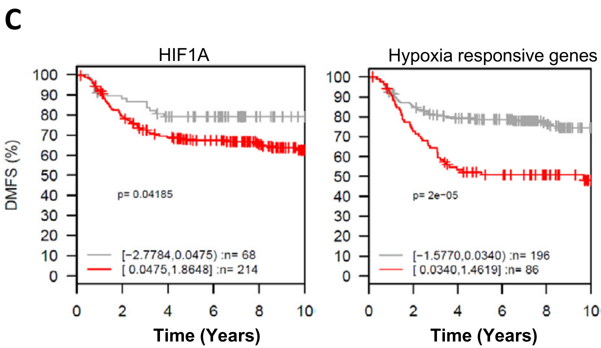

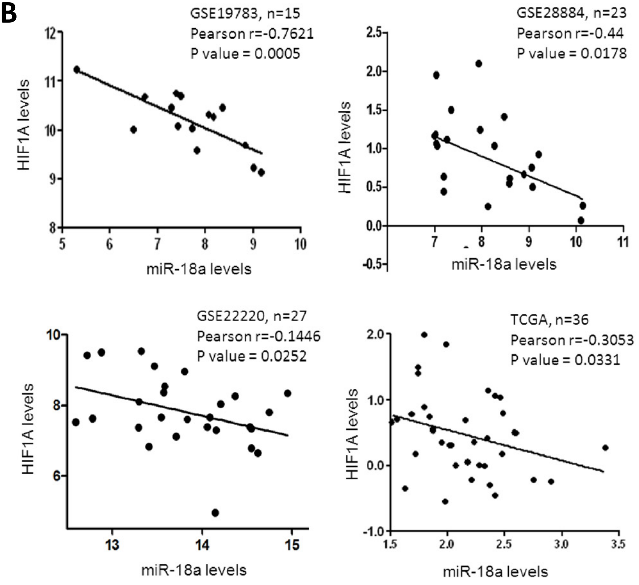

Hypoxia responsive genes negatively correlated with miR-18a in basal-like breast cancer tumors

Gene	Pearson r	P value	Data set	cases
ADM	-0.310	2.90E-03	GSE28884	n=23
AKAP12	-0.519	2.80E-03	GSE22220	n=27
ANG*	-0.306	3.20E-03	GSE28884	n=23
ANGPTL4	-0.204	3.67E-02	GSE28884	n=23
COL5A1*	-0.460	3.00E-03	TCGA	n=36
CYR61*	-0.577	1.21E-02	GSE19783	n=15
DBP	-0.302	3.60E-03	GSE28884	n=23
DHRS3	-0.236	1.86E-02	GSE28884	n=23
EFEMP2	-0.327	1.80E-03	GSE28884	n=23
EGR1*	-0.675	2.90E-03	GSE19783	n=15
FILIP1L*	-0.657	3.90E-03	GSE19783	n=15
FKBP14*	-0.400	8.00E-03	TCGA	n=36
GJA1	-0.281	6.40E-03	GSE28884	n=23
HTRA1	-0.706	1.60E-03	GSE19783	n=15
LOXL1*	-0.501	2.85E-02	GSE19783	n=15
PCOLCE*	-0.420	6.00E-03	TCGA	n=36
PDGFRL*	-0.390	1.00E-02	TCGA	n=36
PHLDA3*	-0.554	1.60E-02	GSE19783	n=15
RAI14*	-0.473	3.77E-02	GSE19783	n=15
SDC2	-0.558	1.54E-02	GSE19783	n=15
SERPINB6	-0.220	2.67E-02	GSE28884	n=23
SLC2A3*	-0.545	1.78E-02	GSE19783	n=15

All listed genes were found to be negatively correlated with miR-18a expression ($P<0.05$) in more than two datasets. The dataset with lowest p value for each gene is presented.

*Genes downregulated by miR-18a in LM cells under hypoxia based on RT-qPCR analysis
